# Novel 2,4-Disubstituted-1,3-Thiazole Derivatives: Synthesis, Anti-*Candida* Activity Evaluation and Interaction with Bovine Serum Albumine

**DOI:** 10.3390/molecules25051079

**Published:** 2020-02-28

**Authors:** Andreea-Iulia Pricopie, Monica Focșan, Ioana Ionuț, Gabriel Marc, Laurian Vlase, Luiza-Ioana Găină, Dan C. Vodnar, Elemer Simon, Gabriel Barta, Adrian Pîrnău, Ovidiu Oniga

**Affiliations:** 1 Department of Pharmaceutical Chemistry, “Iuliu Hațieganu” University of Medicine and Pharmacy, 41 Victor Babeș Street, 400012 Cluj-Napoca, Romania; pricopie.andreea@umfcluj.ro (A.-I.P.); marc.gabriel@umfcluj.ro (G.M.); onigao65@yahoo.com (O.O.); 2 Nanobiophotonics and Laser Microspectroscopy Center, Interdisciplinary Research Institute on Bio-Nano-Sciences, Babes-Bolyai University, Treboniu Laurean No. 42, 400271 Cluj-Napoca, Romania; 3 Department of Pharmaceutical Technology and Biopharmaceutics, “Iuliu Hațieganu” University of Medicine and Pharmacy, 41 Victor Babeș Street, 400012 Cluj-Napoca, Romania; laurian.vlase@umfcluj.ro; 4 Research Center on Fundamental and Applied Heterochemistry, Faculty of Chemistry and Chemical Engineering, “Babeş-Bolyai” University, 11 Arany Janos Street, 400028 Cluj-Napoca, Romania; gaina.ioana.luiza@gmail.com; 5 Department of Food Science and Technology, University of Agricultural Sciences and Veterinary Medicine, 3-5 Mănăștur Street, 400372 Cluj-Napoca, Romania; dan.vodnar@usamvcluj.ro (D.C.V.); simon.elemer@usamvcluj.ro (E.S.); gabriel.barta@usamvcluj.ro (G.B.); 6 National Institute for Research and Development of Isotopic and Molecular Technologies, 67-103 Donath Street, 400293 Cluj-Napoca, Romania; adrian.pirnau@itim-cj.ro

**Keywords:** 1,3-thiazole, anti-*Candida*, cell membrane integrity, fluorescence microscopy, bovine serum albumin

## Abstract

Herein we report the synthesis of two novel series of 1,3-thiazole derivatives having a lipophilic C4-substituent on account of the increasing need for novel and versatile antifungal drugs for the treatment of resistant *Candida sp.*-based infections. Following their structural characterization, the anti-*Candida* activity was evaluated in vitro while using the broth microdilution method. Three compounds exhibited lower Minimum Inhibitory Concentration (MIC) values when compared to fluconazole, being used as the reference antifungal drug. An in silico molecular docking study was subsequently carried out in order to gain more insight into the antifungal mechanism of action, while using lanosterol-C14α-demethylase as the target enzyme. Fluorescence microscopy was employed to further investigate the cellular target of the most promising molecule, with the obtained results confirming its damaging effect towards the fungal cell membrane integrity. Finally, the distribution and the pharmacological potential in vivo of the novel thiazole derivatives was investigated through the study of their binding interaction with bovine serum albumin, while using fluorescence spectroscopy.

## 1. Introduction

Invasive candidiasis rank among the top four causes of nosocomial infectious diseases, being associated with an alarming mortality rate, especially in immunocompromised and critically ill patients [1]. 

*Candida albicans* is the predominant pathogenic strain that is involved in candidaemia. However, the increased antifungal selective pressure, which is mainly mediated by the overuse of fluconazole and other azole drugs, led to a progressive aethiological shift to non-albicans *Candida* sp., such as *C. glabrata* or *C. auris*, together with an increasing resistance to the marketed antifungals [2]. Among the virulence traits of *C. albicans,* is its ability to form polymicrobial biofilms is noteworthy, which enables the development of mixed bacterial and fungal infections that are highly resistant to antimicrobial agents [3,4]. *C. albicans* acts synergistically with some bacterial species, such as methicillin resistant *Staphylococcus aureus* (MRSA), providing protection against the bactericidal effect of antibiotics. Moreover, the fungal biofilm supplies the growth and development of anaerobic strains, such as *Clostridium difficile* and *Bacterioides fragilis*, in external aerobic conditions by creating a protective hypoxic microenvironment [5].

Resistance to antifungal treatment is a serious concern on account of both the limited number of available chemotherapeutic agents and the phylogenetic relatedness between fungal and human eukaryotic cells, which hinder the development of novel active drugs with an acceptable pharmaco-toxicological profile [6]. However, fungi also possess distinct cytoplasmic organelles and biosynthetic pathways [7]. The enzymes that are involved in the biosynthesis of ergosterol are important targets for several clinically approved antifungals. Among these, the most widely used are the azole compounds, which inhibit the CYP51 enzyme-mediated demethylation of lanosterol into ergosterol, which is a major component of the fungal cytoplasmic membrane, being absent in the mammalian cells, and it is also a bioregulator of membrane integrity and proper function of membrane-bound enzymes [8,9]. The resulted disruption of fungal cell membrane morphology and functional integrity, with the subsequent loss of intracellular constituents and cell lysis, is responsible for their fungistatic effect [7]. However, drawbacks, such as narrow antifungal spectrum, low bioavailability, systemic toxicity, and increasing drug tolerance, require the development of novel molecules with improved pharmacokinetics and therapeutic efficiency [10]. 

In the field of antifungal drugs discovery, significant efforts have been made towards the chemical development of molecules containing azole heterocyclic structures as key pharmacophores [11]. 1,3-thiazole and 2-hydrazynil-1,3-thiazole derivatives having a lipophilic C4-substituent are endowed with potent anti-*Candida* activity against clinically relevant fungal strains, as reported in the literature [12,13] and supported by the results that were obtained in our previous research [14]. Thus, we decided to synthesize and evaluate the antifungal potential of two novel series of compounds, keeping the key pharmacophores constant, 1,3-thiazole and 2-hydrazinyl-1,3-thiazole, and introducing different moieties at the C4-position of the thiazole heterocycle. 

The inhibitory activity of the newly synthesized compounds against pathogenic *Candida sp*. strains was evaluated in vitro and the results obtained, being expressed in terms of Minimum Inhibitory Concentration (MIC) and Minimum Fungicidal Concentration (MFC), were compared to those of the reference drug fluconazole. 

A molecular docking study was performed while using fungal lanosterol-C14α-demethylase (CYP51) as a target enzyme in an attempt to gain more insight into the mechanism of action of the novel thiazole derivatives. Molecular docking is widely used for the approximate prediction of the binding affinity of a molecule towards a biological target using scoring functions, owing to the progress in structural analysis techniques and the consequential accessibility to experimentally derived ligand-receptor complexes [15]. 

The most active molecule in both in vitro and in silico antifungal assay was subjected to a fluorescence microscopy study, while using the membrane-impermeable DNA-binding fluorescent dye propidium iodide (PI) as a tracer, in order to confirm the disruptive effect on the integrity of the fungal cell membrane [16].

Protein-drug interaction studies of antifungals can provide a structural guideline and rational drug design, which is helpful in the synthesis of novel molecules with enhanced clinical efficiency and reduced toxicity [17]. The study of the pharmacokinetic profile of the newly synthesized compounds in terms of binding profile to plasmatic carrier proteins, such as human serum albumin (HSA), provides valuable insight into their pharmacological potential in vivo [18]. Generally, a weak binding interaction of a drug with serum albumin is associated with a short lifetime and poor distribution to the target site in vivo, while a strong binding interaction leads to low free drug plasma concentration, which is responsible for the therapeutic effect [19]. Bovine serum albumin (BSA) is widely used as a model protein for these experimental studies due to its high homology with HSA, having 75% identity and 87% similarity shared between amino acid sequences, and its relatively low cost [20]. Herein, fluorescence spectroscopy was employed for the analysis of the novel compounds–BSA molecular interactions, with this analytical method being preferred because of its sensitivity, rapidity, and simplicity [21]. 

## 2. Results and Discussion

### 2.1. Chemistry

**Scheme 1:**
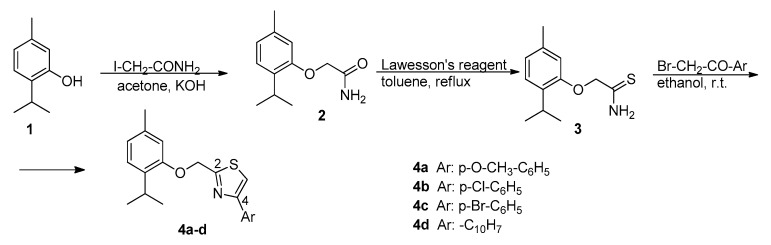
Synthetic protocol of the 4-phenyl-1,3-thiazole derivatives **4a**–**d**.

The novel 4-substituted-1,3-thiazole derivatives (**4a**–**d**) were obtained according to the classical Hantzsch condensation protocol, as illustrated in Scheme 1. The synthesis of the intermediate thioamide **3** was previously reported by our group [14]. 

**Scheme 2:**
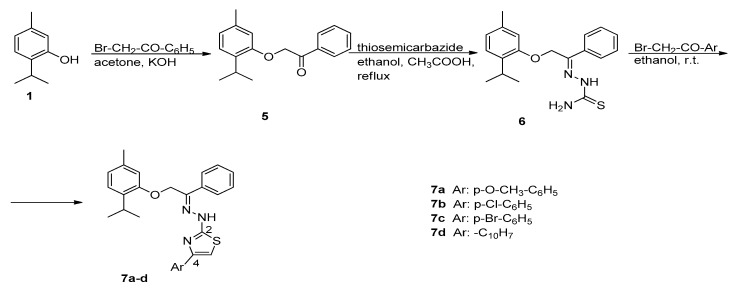
Synthetic protocol of the 2-hydrazinyl-4-phenyl-1,3-thiazole derivatives **7a**–**d**.

The synthetic protocol that was employed for the chemical development of the compounds **7a**–**d** is outlined in Scheme 2. The previously obtained thiosemicarbazone **6** [14] was subjected to a heterocyclization with the corresponding α-haloketones, yielding the targeted 2-hydrazinyl-4-substituted-1,3-thiazole derivatives.

Elemental analysis and spectral data ((Fourier transform infrared spectroscopy (FT-IR), electrospray ionization-mass spectrometry (ESI-MS), proton nuclear magnetic resonance (^1^H-NMR), and carbon nuclear magnetic resonance (^13^C-NMR)) were used for the structural confirmation of the newly synthesized compounds (see Supplementary Material, Figures S1–32). The results of the C, H, N, and S quantitative elemental analysis were consistent with the calculated values, within ± 0.4% of the theoretical values. The recorded molecular ion peaks (M + 1) were consistent with their molecular formulas.

In regard to the first series of compounds (**4a**–**d**), the spectral data confirmed the successful accomplishment of Hantzsch condensation through the appearance of specific thiazole-C5-H singlet at 7.97–8.31 ppm in the ^1^H-NMR spectra, as well as the presence of a characteristic signal in the IR spectra at 3089–3106 cm^−1^, which corresponded to the C5-H stretching vibration.

Concerning the 2-hydrazinyl-thiazole derivatives (**7a**–**d**), two specific signals appeared in their IR spectra, at 3318–3327 cm^−1^ and 3189–3200 cm^−1^, corresponding to the N-H asymmetric and symmetric stretching vibrations. Moreover, the presence of a characteristic signal in the ^13^C-NMR spectra at 143.2–144.1 ppm, belonging to the sp^2^ carbon from hydrazone linker, along with the specific ^1^H-NMR signals that were related to C5-H (7.15–7.50 ppm) and additional aromatic protons, confirmed the proposed structures. 

### 2.2. In Vitro Anti-Candida Activity 

The antifungal activity of the synthesized thiazole derivatives was evaluated in vitro against three human pathogenic *Candida* strains. The stock solutions (1 mg/mL) were prepared by dissolving the tested compounds and the reference antifungal drug, fluconazole, in sterile DMSO. The MIC and MFC values were determined through the broth microdilution method, and they are presented in Table 1 and Table 2.

The obtained MIC and MFC values revealed moderate to very good anti-*Candida* activities for the tested compounds. 

2-Hydrazinyl-thiazole derivatives having a lipophilic (+π) para-substituent in the C4 position of the azole heterocycle (**7a, 7b,** and **7c**) were the most promising, exhibiting MIC values (3.9 μg/mL) that were four times lower when compared to the reference drug fluconazole (15.62 μg/mL) against the pathogenic *Candida albicans* strain. The non-albicans species proved to be less susceptible to almost all of the tested molecules. This findings are in accordance with the results obtained in our previous study [14]. The replacement of the C4-para-substituted-phenyl ring with a naphthyl substituent, in the case of compound **7d**, led to a decrease in the inhibitory activity on both albicans and non-albicans strains.

The obtained data emphasize that the antifungal activity is dependent on an optimal hydro-lipophilic balance. An excessive hydrophobicity and high aromaticity could bring forth an increased drug efflux mediated by the membrane-associated multidrug efflux pumps, and thus a lower biological activity, despite the higher permeability through the fungal cell membrane [22].

While analyzing the obtained results, it seems reasonable to assume that the presence of a hydrazine substituent in the C2 position of the azole heterocycle is correlated with an increased antifungal efficiency, since the MIC values that were obtained for the first series of 4-substituted-1,3-thiazole derivatives (**4a**–**d**) were significantly higher than those recorded for the second series of 4-substituted-2-hydrazinyl-1,3-thiazoles (**7a**–**d**). 

The determination of MFC confirmed the previously obtained MIC values. The MFC/MIC ratio values for all of the tested compounds were equal to 2, which suggested that these molecules may exert a fungicidal effect [23].

### 2.3. Molecular Docking Study

Ergosterol biosynthesis is one of the most important cellular targets employed for the development of novel antifungals, being the main sterol responsible for the fungal cell membrane integrity and function, which is structurally different from the mammalian cholesterol [24,25].

We speculate that the newly synthesized compounds might act through altering the ergosterol biosynthetic pathway due to the promising MIC values obtained for the 2-hydrazinyl-1,3-thiazole derivatives **7a**, **7b**, and **7c**, as compared with the reference drug fluconazole. Aiming to investigate this hypothesis, a molecular docking study was carried out on *Candida albicans* lanosterol-C^14^α-demethylase (CYP51), a validated target in the fungal cell membrane [26].

For each compound, the conformation with the best binding affinity, being expressed as the highest variation of Gibbs free energy (ΔG) of the complex with the biomacromolecule, was predicted by a previously validated ligand-protein interaction protocol [27]. Table 3 presents the computed binding interaction energy (ΔG) and the calculated inhibition constant (ki), together with the analysis of the cluster containing the energetically favorable binding conformations.

The obtained data indicated the existence of binding poses with favorable energies for all of the tested ligands. Concerning the second series of 2-hydrazinyl-4-substituted-1,3-thiazole derivatives (**7a**–**d**), the presence of a hydrazine substituent at the C2 position of the azole heterocycle and the supplementary phenyl ring translate into an improved interaction with the target enzyme in terms of binding energy and steric deviation. However, the increased molecular flexibility that is given out by the C^2^-hydrazine bridge is associated with a larger spatial dispersion of the predicted binding poses as compared with the first series of 4-substituted-1,3-thiazole compounds (**4a**–**d**), as outlined by the increased number of clusters containing residual conformations. 

**Figure 1:**
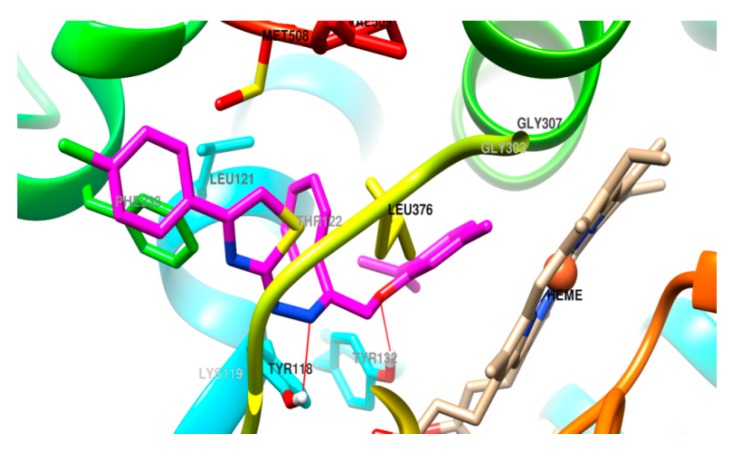
The top binding conformation of compound **7b**. Carbon atoms of the docked compound to the active site of fungal lanosterol C^14^α-demethylase are depicted in magenta. Unnecessary protein fragments in the background and foreground were removed for clarity.

For both series of ligands, the differences in the predicted binding mode and ligand-protein affinity are linked to their electronic, steric, and hydrophobic properties. It can be observed that the binding affinity regularly increased with the lipophilicity of the C4-thiazole substituent. This might be explained by the hydrophobic interactions with the CYP51 lipophilic area that is located in the depth of the binding pocket (Figure 1), as previously reported by our research group [28].

An additional interaction with the polar Tyr132 amino acid sidechain, which is located at the hydrophilic domain of the access channel to the binding site, is due to the presence of the etheric oxygen, which acts as a hydrogen bond acceptor. Additionally, a π-cation interaction is allowed between the thymol fragment and the heme Fe^2+^, due to the parallel orientation of the first one to the catalytic site. 

In regard to the 2-hydrazinyl-4-substituted-1,3 thiazole compounds (**7a**–**d**), a supplementary hydrogen bond is formed between the Tyr118 amino acid sidechain and the hydrazine bridge. 

The results of the molecular docking study suggested that the tested compounds might act as non-competitive inhibitors of the fungal lanosterol C14α-demethylase, interfering with the access and the subsequent binding of the physiological substrate to the catalytic site. The newly synthesized thiazole derivatives might be associated with reduced toxicity and better activity against resistant strains since the mechanism of action is not related to the covalent coordination of the heme Fe^2+^, as in the case of classical antifungal azoles [29,30]. 

### 2.4. Propidium Iodide (PI) Dye Uptake Assay

The PI dye exclusion assay was performed to further support the cell membrane damaging effect of compound **7b**, which exhibited the strongest anti-*Candida* activity in both in vitro and in silico studies. In fact, PI is a DNA-staining dye which upon binding to double stranded nucleic acid gives a red fluorescence when excited by a 480 nm laser [31]. This membrane-impermeant dye is generally excluded from viable cells, unless their membrane integrity is compromised [32].

**Figure 2:**
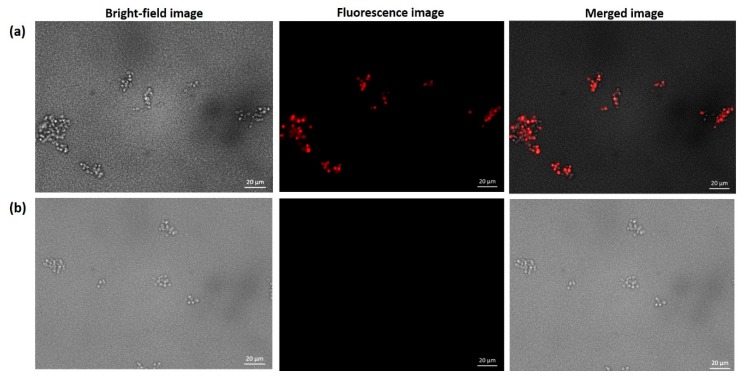
Representative bright-field, fluorescence and merged images of *Candida albicans* cells stained with Propidium Iodide (PI): (**a**) cells exposed to MIC concentration of **7b**; and, (**b**) control cells.

Incubation of *Candida albicans* stained cells with MIC concentration of **7b** resulted in a red fluorescence signal, as evidenced by fluorescence microscopy, as shown in Figure 2a. No fluorescence was observed in the control cells, being incubated with PI alone (Figure 2b).

The obtained results indicate the PI uptake in *C. albicans* cells exposed to the newly synthesized 2-hydrazinyl-1,3-thiazole derivative **7b**, which suggest that this compound might act through the disruption of the fungal cell membrane structural integrity.

### 2.5. Protein Binding Study

BSA is a globular protein, which consists of a single polypeptide chain composed of 583 amino acid residues. At the physiological neutral pH, BSA presents a native form, its tertiary structure comprising three homologous domains (I, II, and III), each being divided into two subdomains (A and B) [20]. The intrinsic fluorescence properties of BSA are mainly due to the existence of two Trp residues. One is located on the surface of the macromolecule (Trp 134) and is more exposed to a hydrophilic environment. The other (Trp 213) is positioned in the hydrophobic pocket of the domain II and it is highly sensitive to ligand binding-induced modifications in the microenvironment and to conformational changes [33]. 

The fluorescence spectra of BSA upon the addition of increasing concentrations (0.3 μM, 0.6 μM, 0.9 μM, 1.2 μM, 1.5 μM, and 1.8 μM) of the tested ligands were recorded in order to analyze the binding profile of compounds **4a**–**d** and **7a**–**d** to serum albumin, in terms of binding mechanism, binding constants and the number of binding sites. Spectral analysis was performed at room temperature under simulated physiological conditions (pH = 7.4), by the excitation of the probes at 290 nm and emission scans ranging between 300 to 450 nm. 

The emission band that is centered at 341 nm (λ_max_) originates from Trp residue. As a first observation, the fluorescent intensity of BSA regularly decreased with the increasing concentration of the tested molecules, which is in good agreement with the classical Stern–Volmer equation (1), which confirmed the binding interaction of the ligands with the biomacromolecule [34].

F0/F = 1 + K_SV_ × [Q] = 1+K_q_ × г_0_ × [Q] or (F0 − F)/F = K_SV_ × [Q] = K_q_ × г_0_ × [Q] (1)

where F0 and F are the fluorescence intensities of BSA in the absence and presence of the quencher, respectively; K_SV_ is the Stern–Volmer quenching constant; and, [Q] represents the concentration of the quencher. K_q_ is the quenching rate constant of the biomolecule and г_0_ is the average fluorescence lifetime of the biomolecule without the quencher, which is approximately 6 ns for BSA. 

**Figure 3:**
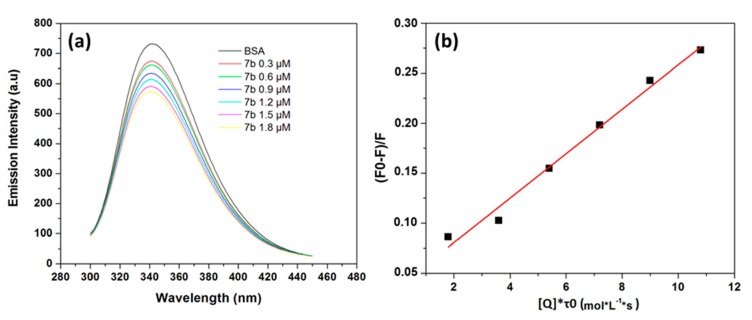
(**a**) Fluorescence spectra of bovine serum albumin (BSA) in the presence of compound **7b**. (**b**) The Stern–Volmer curve for the quenching of BSA (1.5 μM) by increasing concentrations (0.3 μM, 0.6 μM, 0.9 μM, 1.2 μM, 1.5 μM, and 1.8 μM) of **7b** (λex = 289 nm, T = 298K).

Figure 3a illustrates the fluorescence spectra of BSA in the presence of increasing concentrations of **7b**, being selected as the most active compound in both in vitro and in silico antifungal assays. 

The binding region should be in the vicinity of Trp residue, since a distant event cannot cause its fluorescence quenching [35]. No change of the maximum emission wavelength was observed in the case of the 4-phenyl-substituted-1,3 thiazole (**4a**–**c**) and 2-hydrazynil-1,3-thiazole (**7a**–**d**) derivatives. For compound **4d,** having a naphthalene substituent at the C4 position of the thiazole ring, the fluorescence quenching was accompanied by a slight red shift of the λ_max_ (from 341 nm to 345 nm), which suggested the tertiary structure modification of the protein and an increase in the polarity of the microenvironment around the Trp residue due to solvent exposure [21].

The drug binding-induced fluorescence quenching of serum albumin can occur due to either a dynamic or a static mechanism. Molecular collisions between the ligand and the fluorophore in excited state mediate dynamic fluorescence quenching, while the static quenching results from the formation of a ground state fluorophore–ligand complex, thus reducing the population of fluorescent motifs that are capable of excitation [36].

K_SV_ and K_q_, according to Stern–Volmer equation, were determined by the slope and the intercept of the linear regression plot graph of the relative emission intensity (F0−F)/F versus г_0_ × [Q], as illustrated in Figure 3b for compound **7b**. Table 4 presents the calculated values for all of the tested compounds.

The calculated quenching constant (K_q_) of the tested compounds is greater when compared to the value that was obtained for biological macromolecules quenching due to collision mechanism (2 × 10^10^ M^−1^ × s^−1^), suggesting that a static quenching is involved, through the formation of a BSA-ligand complex [33]. 

The K_sv_ value reflects the magnitude of quenching, which is dependent on the ligand molecule’s availability to the fluorophore residue, as well as on the lipophilicity and electronic effects of the C4-thiazole substituent [18]. For compounds **4d** and **7d**, the increased hydrophobicity of the molecule as a whole, due to the presence of naphthalene substituent at C4 position of the thiazole heterocycle, might explain the strength of the interaction with BSA. The presence of two fused aromatic rings results in a higher ring planarity and greater conjugation (π–π interactions) to the hydrophobic pocket of domain II [37].

In the case of a static quenching mechanism, the number of binding sites (n) and binding constants (K_b_) can be determined by the intercept and the slope of the regression curve, while using Equation (2) [38]. Table 5 presents the obtained results for all of the tested compounds.

log[(F0 − F)/F] = log K_b_ + n log[Q] (2)

The K_b_ values reflect the strength of the binding interaction. For the majority of the tested molecules, the K_b_ values are in the range of 1−15 × 10^4^ L/mol, which suggests the existence of a reversible and moderate interaction in BSA-ligand complex, which is associated with a faster diffusion rate in vivo to reach the target site [39]. The calculated n values are approximately 1, indicating the existence of a single binding site of the newly synthesized thiazole derivatives on BSA [40].

## 3. Materials and Methods 

### 3.1. General Information

All of the chemicals (reagent grade) used for synthesis were obtained from commercial sources and used as supplied, without further purification. For the monitoring of the reaction progress and the purity of the newly synthesized compounds, an analytical thin layer chromatography (TLC) carried out on Merck precoated Silica Gel 60F254 sheets (Darmstadt, Germany) was employed. A mixture of ethyl acetate: n-heptane = 3:1 was used as an elution system and the visualization was made while using UV light (254 nm). The melting points were determined with an Electrothermal melting point meter through the open glass capillary method and they are presented uncorrected. The structures of the synthesized compounds were assigned through spectral data (mass spectrometry (MS), infrared spectroscopy (IR), and nuclear magnetic resonance (NMR)) and their purity was confirmed by elemental analysis. The IR spectra were recorded on a Jasco FT/IR 6100 spectrometer (Jasco, Easton, MD), while using anhydrous potassium bromide for sample preparation. The MS analyses were performed in positive ionization, while using an Agilent 1100 series and an Agilent Ion Trapp SL mass spectrometer (Agilent, Santa Clara, CA, USA). The ^1^H-NMR spectra were recorded on a Bruker Advance NMR spectrometer (Karlsruhe, Germany), operating at 500 MHz, while using DMSO-*d*_6_ as solvent and tetramethylsilane (TMS) as internal standard. ^13^C-NMR analyses were performed on a Bruker Advance NMR spectrometer, operating at 125 MHz, in DMSO-*d*_6_, while using a Waltz-16 decoupling scheme, with TMS as the internal standard. Chemical shift (δ) values were reported in parts per million (ppm). Splitting patterns are given as s (singlet), d (doublet), t (triplet), and m (multiplet).

### 3.2. Chemistry

#### 3.2.1. General Procedure for the Synthesis of 2-((2-isopropyl-5-methylphenoxy)methyl)-4-phenyl Thiazole Derivatives 4a–d

Equimolar quantities (1 mmol) of carbothioamide **3** and corresponding α-haloketones were dissolved in dry acetone (3 mL) and stirred at room temperature for 6 h. The resulted precipitate was filtered under vacuum and then washed with a solution of NaHCO_3_ 10% until free of acid. The pure compounds were yielded through recrystallization from ethanol. 

*2-((2-isopropyl-5-methylphenoxy)methyl)-4-(4-methoxyphenyl)thiazole* (**4a**): 0.33 g, yield 63%; m.p. 186–187 °C; FT-IR (KBr) ν_max_ cm^−1^: 3089 (C_5_–H thiazole str), 3048 (C–H_ar_ str), 2921 (C-H_alif_ str), 1612 (C=N str), 1257 (C–O–C asym str), 1050 (C–O–C sym str); ^1^H NMR (500 MHz, DMSO-*d*_6_, δ/ppm): 7.97 (s, 1H, thiazole-C_5_H), 7.90 (d, *J* = 9.0 Hz, 2H, Ar-H), 7.11 (d, *J* = 7.5 Hz, 1H, Ar-H), 7.01 (d, *J* = 9.0 Hz, 2H, Ar-H), 6.95 (s, 1H, Ar-H), 6.78 (d, *J* = 7.5 Hz, 1H, Ar-H), 5.46 (s, 2H, O-CH_2_), 3.80 (s, 3H, Ar-CH_3_), 3.33–3.27 (m, 1H, Ar-CH-(CH_3_)_2_)), 2.27 (s, 3H, Ar-CH_3_), 1.20 (d, *J* = 7.0 Hz, 6H, Ar-CH-(CH_3_)_2_); ^13^C NMR (125 MHz, DMSO-*d*_6_, δ/ppm): 167.5 (C), 159.7 (C), 155.0 (C), 154.5 (C), 136.5 (C), 133.7 (C), 127.8 (2CH), 127.3 (C), 126.3 (CH), 122.4 (CH), 114.6 (2CH), 113.5 (CH), 113.1 (CH), 67.5 (CH_2_), 55.6 (CH_3_), 26.8 (CH), 23.1 (2CH_3_), 21.4 (CH_3_); MS (ESI) *m*/*z*: calculated for C_21_H_23_NO_2_S [M + H]^+^ 354.1, found 354.3; Anal. calculated for C_21_H_23_NO_2_S (%): C, 71.36; H, 6.56; N, 3.96; S, 9.09; found (%): C, 71.49; H, 6.53; N, 3.94; S, 9.07.

*4-(4-chlorophenyl)-2-((2-isopropyl-5-methylphenoxy)methyl)thiazole (***4b***)*: 0.31 g, yield 58%; m.p. 182–183 °C; FT-IR (KBr) ν_max_ cm^−1^: 3106 (C_5_–H thiazole str), 3050 (C–H_ar_ str), 2920 (C-H_alif_ str), 1613 (C=N str), 1256 (C–O–C asym str), 1049 (C–O–C sym str), 745 (C–Cl str); ^1^H NMR (500 MHz, DMSO-*d*_6_, δ/ppm): 8.23 (s, 1H, thiazole-C_5_H), 8.00 (d, *J* = 9.0 Hz, 2H, Ar-H), 7.53 (d, *J* = 9.0 Hz, 2H, Ar-H), 7.12 (d, *J* = 7.5 Hz, 1H, Ar-H), 6.95 (s, 1H, Ar-H), 6.79 (d, *J* = 7.5 Hz, 1H, Ar-H), 5.48 (s, 2H, O-CH_2_), 3.33–3.28 (m, 1H, Ar-CH-(CH_3_)_2_)), 2.27 (s, 3H, Ar-CH_3_), 1.20 (d, *J* = 7.0 Hz, 6H, Ar-CH-(CH_3_)_2_); ^13^C NMR (125 MHz, DMSO-*d*_6_, δ/ppm): 168.2 (C), 154.9 (C), 153.3 (C), 136.5 (2C), 133.7 (C), 133.3 (C), 129.3 (2CH), 128.1 (2CH), 126.3 (CH), 122.4 (CH), 115.9 (CH), 113.5 (CH), 67.4 (CH_2_), 26.8 (CH), 23.1 (2CH_3_), 21.4 (CH_3_); MS (ESI) *m*/*z*: calculated for C_20_H_20_ClNOS [M + H]^+^ 358.1, found 358.5; Anal. calculated for C_20_H_20_ClNOS (%): C, 67.12; H, 5.63; N, 3.91; S, 8.96; Cl, 9.91; found (%): C, 66.98; H, 5.65; N, 3.90; S, 8.99; Cl, 9.88.

*4-(4-bromophenyl)-2-((2-isopropyl-5-methylphenoxy)methyl)thiazole* (**4c**): 0.41 g, yield 69%; m.p. 198–199 °C; FT-IR (KBr) ν_max_ cm^−1^: 3101 (C_5_–H thiazole str), 3046 (C–H_ar_ str), 2919 (C-H_alif_ str), 1614 (C=N str), 1255 (C–O–C asym str), 1049 (C–O–C sym str), 676 (C–Br str); ^1^H NMR (500 MHz, DMSO-*d*_6_, δ/ppm): 8.24 (s, 1H, thiazole-C_5_H), 7.94 (d, *J* = 9.0 Hz, 2H, Ar-H), 7.67 (d, *J* = 9.0 Hz, 2H, Ar-H), 7.12 (d, *J* = 7.5 Hz, 1H, Ar-H), 6.95 (s, 1H, Ar-H), 6.79 (d, *J* = 7.5 Hz, 1H, Ar-H), 5.48 (s, 2H, O-CH_2_), 3.33–3.28 (m, 1H, Ar-CH-(CH_3_)_2_)), 2.27 (s, 3H, Ar-CH_3_), 1.20 (d, *J* = 7.0 Hz, 6H, Ar-CH-(CH_3_)_2_); ^13^C NMR (125 MHz, DMSO-*d*_6_, δ/ppm): 168.2 (C), 154.9 (C), 153.3 (C), 136.5 (2C), 133.7 (C), 132.29 (2CH), 128.4 (2CH), 126.3 (CH), 122.4 (CH), 121.7 (C), 116.0 (CH), 113.5 (CH), 67.4 (CH_2_), 26.8 (CH), 23.1 (2CH_3_), 21.4 (CH_3_); MS (ESI) *m*/*z*: calculated for C_20_H_20_BrNOS [M + H]^+^ 402.0, found 402.8; Anal. calculated for C_20_H_20_BrNOS (%): C, 59.70; H, 5.01; N, 3.48; S, 7.97; Br, 19.86; found (%): C, 59.88; H, 4.98; N, 3.50; S, 7.94; Br, 19.91.

*2-((2-isopropyl-5-methylphenoxy)methyl)-4-(naphthalen-2-yl)thiazole* (**4d**): 0.39 g, yield 71%; m.p. 179–180 °C; FT-IR (KBr) ν_max_ cm^−1^: 3103 (C_5_–H thiazole str), 3049 (C–H_ar_ str), 2921 (C-H_alif_ str), 1610 (C=N str), 1257 (C–O–C asym str), 1039 (C–O–C sym str); ^1^H NMR (500 MHz, DMSO-*d*_6_, δ/ppm): 8.55 (s, 1H, Ar-H), 8.31 (s, 1H, thiazole-C_5_H), 8.13 (d, *J* = 8.5 Hz, 1H, Ar-H), 8.00 (d, *J* = 8.5 Hz, 2H, Ar-H), 7.95 (d, *J* = 8.5 Hz, 1H, Ar-H), 7.55 (t, 2H, Ar-H), 7.13 (d, *J* = 7.5 Hz, 1H, Ar-H), 6.98 (s, 1H, Ar-H), 6.80 (d, *J* = 7.5 Hz, 1H, Ar-H), 5.53 (s, 2H, O-CH_2_), 3.36–3.31 (m, 1H, Ar-CH-(CH_3_)_2_)), 2.29 (s, 3H, Ar-CH_3_), 1.22 (d, *J* = 7.5 Hz, 6H, Ar-CH-(CH_3_)_2_); ^13^C NMR (125 MHz, DMSO-*d*_6_, δ/ppm): 168.1 (C), 155.0 (C), 154.5 (C), 136.6 (2C), 133.7 (C), 133.1 (C), 131.8 (C), 128.9 (CH), 128.7 (CH), 128.1 (CH), 127.0 (CH), 126.8 (CH), 126.3 (CH), 125.1 (CH), 124.6 (CH), 122.4 (CH), 115.7 (CH), 113.5 (CH), 67.5 (CH_2_), 26.8 (CH), 23.1 (2CH_3_), 21.4 (CH_3_); MS (ESI) *m*/*z*: calculated for C_24_H_23_NOS [M + H]^+^ 374.1, found 374.2; anal. calculated for C_24_H_23_NOS (%): C, 77.18; H, 6.21; N, 3.75; S, 8.58; found (%): C, 77.31; H, 6.19; N, 3.76; S, 8.55.

#### 3.2.2. General Procedure for the Synthesis of 2-(2-(2-(2-isopropyl-5-methylphenoxy)-1-phenyl ethylidene)hydrazineyl)-4-phenylthiazole Derivatives 7a–d

To a solution of hydrazinyl-1-carbothioamide **6** (1 mmol) that was dissolved in dry acetone (3mL), equimolar quantities of corresponding α-haloketones were added and the mixture was stirred at room temperature for 6h. The resulted precipitate was filtered under vacuum and then washed with a solution of NaHCO_3_ 10% until free of acid. The pure compounds were yielded through recrystallization from ethanol. 

*2-(2-(2-(2-isopropyl-5-methylphenoxy)-1-phenylethylidene)hydrazineyl)-4-(4-methoxyphenyl)thiazole* (**7a**): 0.50 g, yield 72%; m.p. 195–196 °C; FT-IR (KBr) ν_max_ cm^−1^: 3327 (N–H asym str), 3194 (N–H sym str), 3106 (C_5_–H thiazole str), 3050 (C–H_ar_ str), 2917 (C-H_alif_ str), 1608 (C=N str), 1253 (C–O–C asym str), 1062 (C–O–C sym str); ^1^H NMR (500 MHz, DMSO-*d*_6_, δ/ppm): 11.44 (br, 1H, NH), 7.79 (d, *J* = 8.8 Hz, 2H, Ar-H), 7.76 (d, *J* = 8.0 Hz, 2H, Ar-H), 7.42 (t, *J* = 7.0 Hz, 2H, Ar–H), 7.37 (t, *J* = 7.0 Hz, 1H, Ar–H), 7.15 (s, 1H, thiazole-C_5_H), 7.05 (d, *J* = 7.5 Hz, 1H, Ar-H), 6.98 (d, *J* = 8.8 Hz, 2H, Ar-H), 6.92 (s, 1H, Ar-H), 6.75 (d, *J* = 7.5 Hz, 1H, Ar-H), 5.30 (s, 2H, O-CH_2_), 3.79 (s, 3H, Ar-CH_3_), 3.05–3.00 (m, 1H, Ar-CH-(CH_3_)_2_)), 2.28 (s, 3H, Ar-CH_3_), 1.00 (d, *J* = 7.0 Hz, 6H, Ar-CH-(CH_3_)_2_); ^13^C NMR (125 MHz, DMSO-*d*_6_, δ/ppm): 170.1 (C), 159.3 (C), 155.2 (C), 143.2 (C), 136.6 (C), 136.2 (2C), 133.6 (2C), 129.3 (CH), 128.7 (2CH), 127.3 (2CH), 126.7 (2CH), 126.1 (CH), 121.8 (CH), 114.5 (2CH), 113.1 (CH), 112.6 (CH), 61.1 (CH_2_), 55.6 (CH_3_) 26.3 (CH), 23.1 (2CH_3_), 21.5 (CH_3_); MS (ESI) *m*/*z*: calculated for C_28_H_29_N_3_O_2_S [M + H]^+^ 472.2, found 472.7; Anal. calculated for C_28_H_29_N_3_O_2_S (%): C, 71.31; H, 6.20; N, 8.91; S, 6.80; found (%): C, 71.52; H, 6.18; N, 8.89; S, 6.82.

*4-(4-chlorophenyl)-2-(2-(2-(2-isopropyl-5-methylphenoxy)-1-phenylethylidene)hydrazineyl)thiazole* (**7b**): 0.54 g, yield 76%; m.p. 190–191 °C; FT-IR (KBr) ν_max_ cm^−1^: 3318 (N–H asym str), 3189 (N–H sym str), 3108 (C_5_–H thiazole str), 3053 (C–H_ar_ str), 2920 (C-H_alif_ str), 1606 (C=N str), 1246 (C–O–C asym str), 1060 (C–O–C sym str), 750 (C–Cl str); ^1^H NMR (500 MHz, DMSO-*d*_6_, δ/ppm): 12.21 (br, 1H, NH), 7.88 (d, *J* = 8.5 Hz, 2H, Ar-H), 7.75 (d, *J* = 7.0 Hz, 2H, Ar-H), 7.56–7.51 (m, 1H, Ar-H), 7.48 (d, *J* = 8.5 Hz, 1H, Ar-H), 7.44 (s, 1H, thiazole-C_5_H), 7.42 (t, *J* = 7.0 Hz, 2H, Ar–H), 7.37 (t, *J* = 7.0 Hz, 1H, Ar–H), 7.04 (d, *J* = 7.5 Hz, 1H, Ar-H), 6.91 (s, 1H, Ar-H), 6.74 (d, *J* = 7.5 Hz, 1H, Ar-H), 5.28 (s, 2H, O-CH_2_), 3.03–2.97 (m, 1H, Ar-CH-(CH_3_)_2_)), 2.28 (s, 3H, Ar-CH_3_), 0.99 (d, *J* = 6.5 Hz, 6H, Ar-CH-(CH_3_)_2_); ^13^C NMR (125 MHz, DMSO-*d*_6_, δ/ppm): 168.8 (C), 155.2 (C), 151.2 (C), 143.6 (C), 136.5 (C), 136.2 (C), 133.5 (C), 132.5 (C), 130.4 (C), 129.1 (2CH), 128.7 (2CH), 127.6 (2CH), 126.7 (2CH), 126.1 (CH), 121.8 (CH), 113.1 (CH), 113.0 (CH), 61.1 (CH_2_), 26.3 (CH), 23.1 (2CH_3_), 21.5 (CH_3_); MS (ESI) *m*/*z*: calculated for C_27_H_26_ClN_3_OS [M + H]^+^ 476.1, found 476.5; Anal. calculated for C_27_H_26_ClN_3_OS (%): C, 68.12; H, 5.51; N, 8.83; S, 6.73; Cl, 7.45; found (%): C, 68.34; H, 5.52; N, 8.81; S, 6.75; Cl, 7.46.

*4-(4-bromophenyl)-2-(2-(2-(2-isopropyl-5-methylphenoxy)-1-phenylethylidene)hydrazineyl)thiazole* (**7c**): 0.52 g, yield 67%; m.p. 187–188 °C; FT-IR (KBr) ν_max_ cm^−1^: 3318 (N–H asym str), 3192 (N–H sym str), 3113 (C_5_–H thiazole str), 3053 (C–H_ar_ str), 2919 (C-H_alif_ str), 1604 (C=N str), 1245 (C–O–C asym str), 1071 (C–O–C sym str), 685 (C–Br str); ^1^H NMR (500 MHz, DMSO-*d*_6_, δ/ppm): 11.99 (br, 1H, NH), 7.82 (d, *J* = 8.5 Hz, 2H, Ar-H), 7.76 (d, *J* = 6.5 Hz, 2H, Ar-H), 7.62 (d, *J* = 8.5 Hz, 2H, Ar-H), 7.46 (s, 1H, thiazole-C_5_H), 7.42 (t, *J* = 7.0 Hz, 2H, Ar–H), 7.38 (t, *J* = 7.0 Hz, 1H, Ar–H), 7.05 (d, *J* = 7.5 Hz, 1H, Ar-H), 6.91 (s, 1H, Ar-H), 6.75 (d, *J* = 7.5 Hz, 1H, Ar-H), 5.28 (s, 2H, O-CH_2_), 3.04–2.98 (m, 1H, Ar-CH-(CH_3_)_2_)), 2.28 (s, 3H, Ar-CH_3_), 0.99 (d, *J* = 7.0 Hz, 6H, Ar-CH-(CH_3_)_2_); ^13^C NMR (125 MHz, DMSO-*d*_6_, δ/ppm): 169.2 (C), 155.2 (C), 151.7 (C), 143.6(C), 136.5 (C), 136.2 (C), 133.6 (C), 133.5 (C), 132.08 (2CH), 129.2 (CH), 128.8 (2CH), 128.0 (2CH), 126.7 (2CH), 126.1 (CH), 121.8 (CH), 121.1 (C), 113.0 (CH), 111.9 (CH), 61.1 (CH_2_), 26.3 (CH), 23.1 (2CH_3_), 21.5 (CH_3_); MS (ESI) *m*/*z*: calculated for C_27_H_26_BrN_3_OS [M + H]^+^ 520.1, found 521.1; Anal. calculated for C_27_H_26_BrN_3_OS (%): C, 62.31; H, 5.04; N, 8.07; S, 6.16; Br, 15.35; found (%): C, 62.19; H, 5.05; N, 8.10; S, 6.14; Br 15.31.

*2-(2-(2-(2-isopropyl-5-methylphenoxy)-1-phenylethylidene)hydrazineyl)-4-(naphthalen-2-yl)thiazole* (**7d**): 0.45 g, yield 61%; m.p. 183–184 °C; FT-IR (KBr) ν_max_ cm^−1^: 3326 (N–H asym str), 3200 (N–H sym str), 3089 (C_5_–H thiazole str), 3050 (C–H_ar_ str), 2922 (C-H_alif_ str), 1605 (C=N str), 1256 (C–O–C asym str), 1091 (C–O–C sym str); ^1^H NMR (500 MHz, DMSO-*d*_6_, δ/ppm): 12.02 (br, 1H, NH), 8.41 (s, 1H, Ar-H), 8.02 (d, *J* = 9.0 Hz, 1H, Ar-H), 7.95 (d, *J* = 9.0 Hz, 1H, Ar-H), 7.92 (d, *J* = 9.0 Hz, 2H, Ar-H), 7.77 (d, *J* = 7.0 Hz, 2H, Ar-H), 7.52 (t, 2H, Ar-H), 7.50 (s, 1H, thiazole-C_5_H), 7.43 (t, *J* = 7.0 Hz, 2H, Ar–H), 7.39 (t, *J* = 7.0 Hz, 1H, Ar–H), 7.06 (d, *J* = 7.5 Hz, 1H, Ar-H), 6.94 (s, 1H, Ar-H), 6.76 (d, *J* = 7.5 Hz, 1H, Ar-H), 5.32 (s, 2H, O-CH_2_), 3.05–3.00 (m, 1H, Ar-CH-(CH_3_)_2_)), 2.30 (s, 3H, Ar-CH_3_), 1.01 (d, *J* = 6.5 Hz, 6H, Ar-CH-(CH_3_)_2_); ^13^C NMR (125 MHz, DMSO-*d*_6_, δ/ppm): 168.5 (C), 155.2 (C), 154.6 (C), 144.1 (C), 136.6 (2C), 136.2 (C), 133.5 (2C), 132.9 (C), 129.2 (CH), 128.8 (2CH), 128.6 (2CH), 128.0 (CH), 126.9 (CH), 126.7 (2CH), 126.5 (CH), 126.1 (CH), 124.5 (CH), 124.3 (CH), 121.8 (CH), 113.1 (CH), 111.8 (CH), 61.1 (CH_2_), 26.3 (CH), 23.1 (2CH_3_), 21.5 (CH_3_); MS (ESI) *m*/*z*: calculated for C_31_H_29_N_3_OS [M + H]^+^ 492.2, found 492.4; Anal. calculated for C_31_H_29_N_3_OS (%): C, 75.73; H, 5.95; N, 8.55; S, 6.52; found (%): C, 75.92; H, 5.93; N, 8.58; S, 6.50.

### 3.3. In Vitro Anti-Candida Activity 

The in vitro anti-*Candida* screening was completed according to the guidelines of Clinical Laboratory Standards Institute (CLSI) [41], with the broth microdilution method being employed for the determination of MIC and MFC values. All of the used fungal strains were obtained from the Food Biotechnology Laboratory, Life Sciences Institute, University of Agricultural Sciences and Veterinary Medicine Cluj-Napoca, Romania. 

Potato dextrose agar medium (Sifin, Germany) was used for the storage of the standardized cell cultures and Roswell Park Memorial Institute (RPMI) 1640 medium with L-glutamine, being adjusted to pH 7.0 with 3-(*N*-morpholino) propanesulfonic acid, was used for the susceptibility testing. Prior to antifungal susceptibility evaluation, each strain was inoculated on potato dextrose agar plates to ensure optical growth characteristics and purity. Subsequently, the yeast cells were suspended in saline and adjusted spectrophotometrically to RPMI 1640 medium. The initial density of *Candida sp.* was approximately 2 × 10^6^ colony forming units (CFU)/mL. Inoculums (density of 0.5 in McFarland scale) were prepared in a sterile solution of 0.9% NaCl. Subsequently, the tested strains were suspended in nutrient broth and RPMI 1640 media to achieve a final density of 2 × 10^5^ CFU/mL.

The stock solutions (1 mg/mL) were obtained by dissolving the newly synthesized compounds and the reference antifungal, fluconazole, in sterile DMSO, and they were kept at 4 °C. A series of double diluting solutions of the tested compounds were prepared in RPMI 1640 medium to achieve a final concentrations in the range of 500–0.015 μg/mL.

Media (100 μL) was placed into each 96 wells of the microplates for the determination of MIC/MFC according to broth microdilution method. Sample solutions (100 μL) at high concentration (500 μg/mL) were added into the first column of the microplates and two-fold dilutions of the compounds were obtained by the serial pipetting of the mixed solution (100 μL) into the remaining wells. Ten-microliter culture suspensions were inoculated into all of the wells and the sealed microplates were incubated at 37 °C for 18 h. Growth control, sterility control, and control of the antifungal compounds were used. The plates were incubated at 25 °C for 48 h. Next, minimum inhibitory concentration (MIC) values were spectrophotometrically determined, by recording the optical density test solutions at 600 nm, after the addition of 20 μL of resazurin solution (0.02%). The MIC was defined as the lowest concentration required arresting the growth of the fungi. 0.01 mL of the medium withdrawn from the culture tubes showing no macroscopic growth at the end of the 24 h was sub-cultured on potato dextrose agar plates to determine the number of vital organisms and then incubated further at 37 °C and 25 °C for 24 h and 48 h, respectively, for the determination of the minimum fungicidal concentration (MFC). The MFC was defined as the lowest concentration of the tested compounds at which no fungal colonies were observed. All the MIC and MFC measurements were made in triplicate.

### 3.4. Molecular Docking Study

The binding affinity of the newly synthesized thiazole derivatives (**4a**–**d**, **7a**–**d**) to the catalytic site of the target fungal lanosterol C^14^α-demethylase (CYP51) was evaluated through a molecular docking study, while using AutoDock 4.2.6 [27]. 

The target enzyme was constructed through homology modeling that was based on the UniProt P10613 sequence from *Candida albicans* [42], while using SWISS-MODEL [43]. The homologous sequence that was used as template for the construction of the target macromolecule (PBD 5EQB) was chosen from Protein Data Bank based on the results of a BLAST search. The docking protocol was performed, as previously reported [14]. 

The inhibition constant (Ki) values were calculated based on the in silico predicted binding (∆G), while using the following formula: Ki = e^((∆G × 1000)/(R × T)) (R represents the Regnault constant = 198,719 kcal/(K × mol) and T = 298.15 K).

Visualization and analysis of the docking results were performed while using UCSF Chimera 1.10.2 [44].

### 3.5. Propidium Iodide (PI) Dye Uptake Assay

Fluorescence microscopy was used to evaluate the membrane integrity of yeast cells that were exposed to compound **7b**. For this purpose, *C. albicans* cell suspension (1 × 10^7^ cells/mL) that was prepared in sterile PBS was treated with MIC concentration of **7b** and then incubated for two hours at 37 °C. Cells that were incubated in similar conditions without compound served as the negative control. 

The suspensions were then centrifugated and the cell pellets were washed and resuspended in sterile PBS. PI solution was added to resuspended cells to achieve a final concentration of 1 μg/mL and the samples were incubated for another 30 minutes in the dark. 

The cells were further analyzed with an inverted Zeiss Axio Observer Z1 microscope with a LD Plan Neofluar 20x objective (NA = 0.4, Zeiss). Concretely, the fluorescence images were collected using a Compact Light Source HXP 120 C mercury lamp, the light being reflected by a dichroic mirror while using an excitation filter BP 525/50. An AxioCam Icc digital camera was employed to capture the images, which were then processed while using the ZEN software.

### 3.6. Protein Binding Study

BSA fraction V was purchased from Merck (Darmstadt, Germany) and used as supplied, without further purification. The other used chemicals were of analytical grade purity.

All of the fluorescence emission spectra were recorded on a Jasco FP-6500 spectrofluorometer that was equipped with a DC-powered 150 W Xenon lamp. The preparation of the probes was done according to a previously reported protocol [14]. The BSA concentration was kept constant (1.5 μM) and the concentration of the newly synthesized thiazole derivatives was gradually increased from 0.3 μM to 1.8 μM. The fluorescence emission spectra of BSA-ligand solutions were recorded from 300 to 450 nm, with the excitation wavelength at 290 nm and slid widths of 3 nm. Quartz cells with 1.0 cm path length were employed for the spectral analysis. 

It is worth mentioning that the tested compounds have no fluorescence at the emission wavelength (341 nm). Moreover, they have no absorption at the excitation and emission wavelengths, at the used concentrations, so their inner effect on BSA fluorescence intensity is negligible [19,21]. 

## 4. Conclusions

Altogether, two novel series of thiazole derivatives were synthesized according to a previously reported protocol, which is characterized by physicochemical properties and evaluated in vitro for their anti-*Candida* activity. Three compounds, **7a**, **7b**, and **7c**, exhibited promising results in terms of MIC and MFC. 

According to the data that were obtained from the performed molecular docking study, the newly synthesized compounds might interfere with the ergosterol biosynthetic pathway through a non-competitive inhibition of the fungal lanosterol-C14α-demethylase. *Candida* cells were incubated with the most active molecule, **7b**, and then subjected to a fluorescence microscopy study to further support the proposed hypothesis. PI staining dye uptake, as shown by the captured fluorescence microscopy images, indicated that the tested compound damages the fungal cell membrane integrity.

Additionally, fluorescence spectroscopy studied the affinity of the novel synthesized thiazole derivatives towards carrier plasma proteins, with the obtained results suggesting a moderate and reversible binding interaction with bovine serum albumin, being mediated by the formation of a ground-state complex. 

The present research provides comprehensive information on the development of novel antifungals that are endowed with improved therapeutic efficiency and pharmaco-toxicological profile.

## Figures and Tables

**Table 1:** The minimum inhibitory concentration values (MIC, μg/mL) of the newly synthesized 4-phenyl-1,3-thiazole (**4a**–**d**) and 2-hydrazinyl-4-phenyl-1,3-thiazole (**7a**–**d**) derivatives.

Compound	*C. albicans* ATCC 10231	*C. parapsilosis* ATCC 22019	*C. zeylanoides* ATCC 201082
4a	62.5	62.5	125
4b	62.5	62.5	125
4c	62.5	62.5	125
4d	62.5	62.5	62.5
7a	3.9	15.62	15.62
7b	3.9	15.62	15.62
7c	3.9	15.62	15.62
7d	62.5	62.5	125
Fluconazole	15.62	7.81	15.62
Broth control	No growth		

**Table 2:** The minimum fungicidal concentration values (MFC, μg/mL) of the newly synthesized 4-phenyl-1,3-thiazole (**4a**–**d**) and 2-hydrazinyl-4-phenyl-1,3-thiazole (**7a**–**d**) derivatives.

**Compound**	***C. albicans*** **ATCC 10231**	***C. parapsilosis*** **ATCC 22019**	***C. zeylanoides*** **ATCC 201082**
4a	125	125	250
4b	125	125	250
4c	125	125	250
4d	125	125	125
7a	7.8	31.24	31.24
7b	7.8	31.24	31.24
7c	7.8	31.24	31.24
7d	125	125	250
Fluconazole	31.24	15.62	31.24
Broth control	No growth		

**Table 3:** The predicted binding interaction energy (ΔG, kcal/mol), the consequent inhibition constant (Ki, nM) and the cluster analysis containing the best conformation of each compound given by AutoDock.

Compound	Best Binding Conformation		The 2 Å Cluster Containing the Top Binding Conformation				Number of Distinct Clusters	
	ΔG (kcal/mol)	Ki (nM)	NoC^1^	Average ΔG (kcal/mol)	Standard Deviation			
					ΔG (kcal/mol)	Cartesian Coordinates	Total	Multi-Member
**4a**	−10.87	10.77	30	−10.24	0.49	0.59	20	14
**4b**	−11.17	6.49	24	−10.61	0.56	0.50	22	13
**4c**	−11.45	4.05	29	−10.48	0.52	0.54	19	11
**4d**	−12.36	0.87	17	−11.38	0.57	0.45	27	16
**7a**	−12.63	0.55	10	−11.90	0.46	0.67	48	19
**7b**	−13.16	0.74	10	−11.95	0.45	0.65	54	23
**7c**	−12.46	0.23	4	−12.54	0.44	0.90	49	15
**7d**	−13.61	0.11	9	−13.05	0.64	0.61	47	16

^1^ = number of conformations.

**Table 4:** Stern–Volmer equation constants for the interaction of BSA with the compounds **4a**–**d** and **7a**–**d.**

Compound	K_q_ × 10^12^ (L/mol·s)	^a^ K_SV_ × 10^4^ (L/mol)	^b^ R^2^
**4a**	8.59 ± 0.0004	5.15± 0.0034	0.9838
**4b**	4.94 ± 0.0001	2.94 ± 0.0008	0.9986
**4c**	3.41 ± 0.0001	2.04 ± 0.0011	0.9877
**4d**	17.77 ± 0.0008	10.66 ± 0.0059	0.9899
**7a**	9.36 ± 0.0003	5.58 ± 0.0026	0.9918
**7b**	22.21 ± 0.0012	13.32 ± 0.0086	0.9847
**7c**	2.35 ± 0.0001	1.41 ± 0.0011	0.9872
**7d**	12.75 ± 0.0007	7.65 ± 0.0051	0.9834

^a^ K_SV_ = K_q_× г_0__,_ г_0__≈_ 6 ns; ^b^ R^2^ is the correlation coefficient.

**Table 5:** Binding constant values and number of binding sites for the compounds **4a**–**d** and **7a**–**d** into BSA.

Compound	n	logK_b_	K_b_ × 10^4^ (M^−1^)	R^2^
**4a**	0.773 ± 0.0327	−1.1801 ± 0.0087	6.60	0.9911
**4b**	0380 ± 0.0276	−1.1559 ± 0.0073	6.98	0.9740
**4c**	0.796 ± 0.0260	−1.5819 ± 0.0070	2.61	0.9944
**4d**	1.305 ± 0.0690	−1.0696 ± 0.0186	8.51	0.9859
**7a**	0.987 ± 0.0360	−1.2482 ± 0.0096	5.64	0.9933
**7b**	0.642 ± 0.0445	−0.7441 ± 0.0122	18.02	0.9810
**7c**	0.696 ± 0.0575	−1.7314 ± 0.0158	1.85	0.9732
**7d**	0.912 ± 0.0623	−1.0390 ± 0.0160	9.14	0.9770

## Supplementary Materials

The following are available online, Figures S1–32, containing structural information related to compounds **4a**–**d** and **7a**–**d**.
