# Supplementary material for: Novel 2,4-Disubstituted-1,3-Thiazole Derivatives: Synthesis, Anti-Candida Activity Evaluation and Interaction with Bovine Serum Albumine
Source: Molecules. 2020 Feb 28;25(5):1079. doi: 10.3390/molecules25051079 (PMC7179180; doi:10.3390/molecules25051079)
Supplement: Supplementary file 1 [file molecules-25-01079-s001.pdf]

Supplementary material

Novel 2,4-Disubstituted-1,3-Thiazole Derivatives: Synthesis, Anti-*Candida* Activity Evaluation and Interaction with Bovine Serum Albumine

Andreea-Iulia Pricopie [1], Monica Focșan [2,] *, Ioana Ionuț [1,*], Gabriel Marc [1], Laurian Vlase [3], Luiza-Ioana Găină [4], Dan C. Vodnar [5], Simon Elemer [5], Gabriel Barta [5], Adrian Pîrnău [6] and Ovidiu Oniga [1]

1 Department of Pharmaceutical Chemistry, "Iuliu Hațieganu" University of Medicine and Pharmacy, 41 Victor Babeș Street, 400012 Cluj-Napoca, Romania; pricopie.andreea@umfcluj.ro (A.I.P.), marc.gabriel@umfcluj.ro (G.M.), onigao65@yahoo.com (O.O.);

2 Nanobiophotonics and Laser Microspectroscopy Center, Interdisciplinary Research Institute on Bio-Nano-Sciences, Babes-Bolyai University, Treboniu Laurean No. 42, Cluj-Napoca 400271, Romania; monica.iosin@phys.ubbcluj.ro (M.F.);

3 Department of Pharmaceutical Technology and Biopharmaceutics, "Iuliu Hațieganu" University of Medicine and Pharmacy, 41 Victor Babeș Street, 400012 Cluj-Napoca, Romania; laurian.vlase@umfcluj.ro (L.V.);

4 Research Center on Fundamental and Applied Heterochemistry, Faculty of Chemistry and Chemical Engineering, "Babeş-Bolyai" University, 11 Arany Janos Street, 400028 Cluj-Napoca, Romania; gaina.ioana.luiza@gmail.com (L.G.);

5 Department of Food Science and Technology, University of Agricultural Sciences and Veterinary Medicine, 3-5 Mănăștur Street, 400372 Cluj-Napoca, Romania; dan.vodnar@usamvcluj.ro (D.V.); simon.elemer@usamvcluj.ro (S.E.); gabriel.barta@usamvcluj.ro (G.B.);

6 National Institute for Research and Development of Isotopic and Molecular Technologies, 67-103 Donath Street, 400293 Cluj-Napoca, Romania; adrian.pirnau@itim-cj.ro (A.P);

* Correspondence: monica.iosin@phys.ubbcluj.ro (M.F.); Tel.: +0264454554 (int. 117) (M.F.); ionut.ioana@umfcluj.ro (I.I.); Tel.: +40747-507-629 (I.I.);

Table of contents

Figure 1–8: Copies of ^{1}H-NMR spectra of compounds **4a**–**d** and **7a**–**d**
Figure 9–16: Copies of ^{13}C-NMR spectra of compounds **4a**–**d** and **7a**–**d**
Figure 17–24: Copies of FT-IR spectra of compounds **4a**–**d** and **7a**–**d**
Figure 25–32: Copies of MS analysis of compounds **4a**–**d** and **7a**–**d**

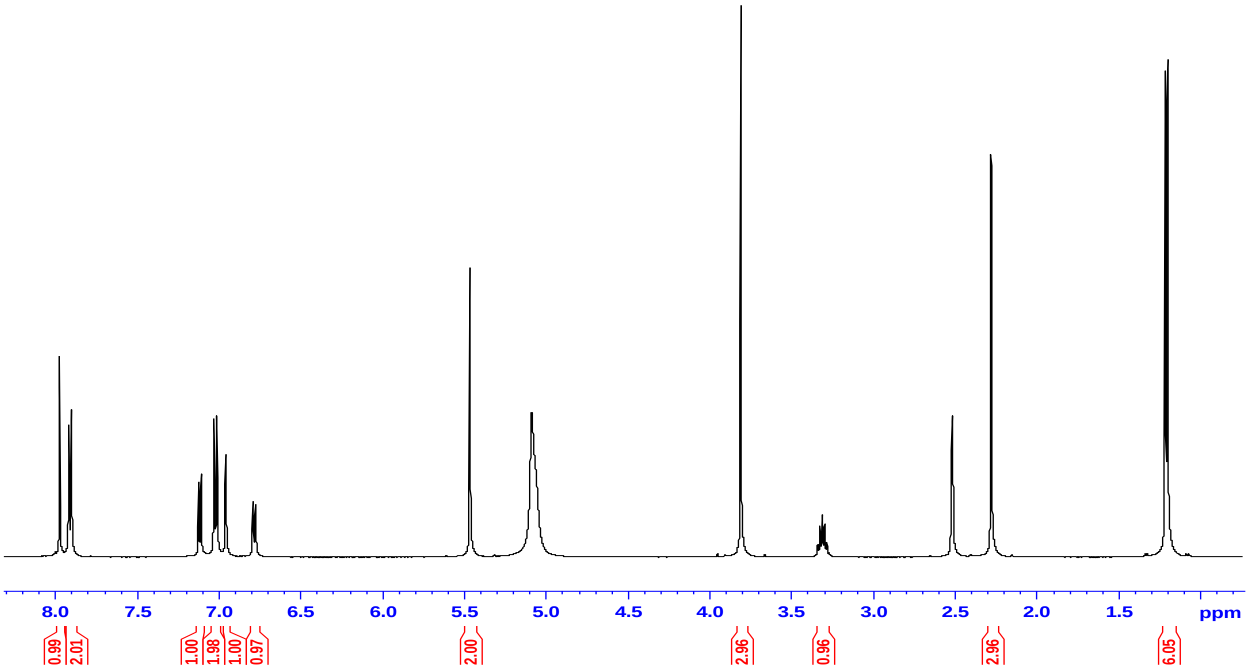

Figure 1. ^{1}H-NMR spectra of compound **4a**

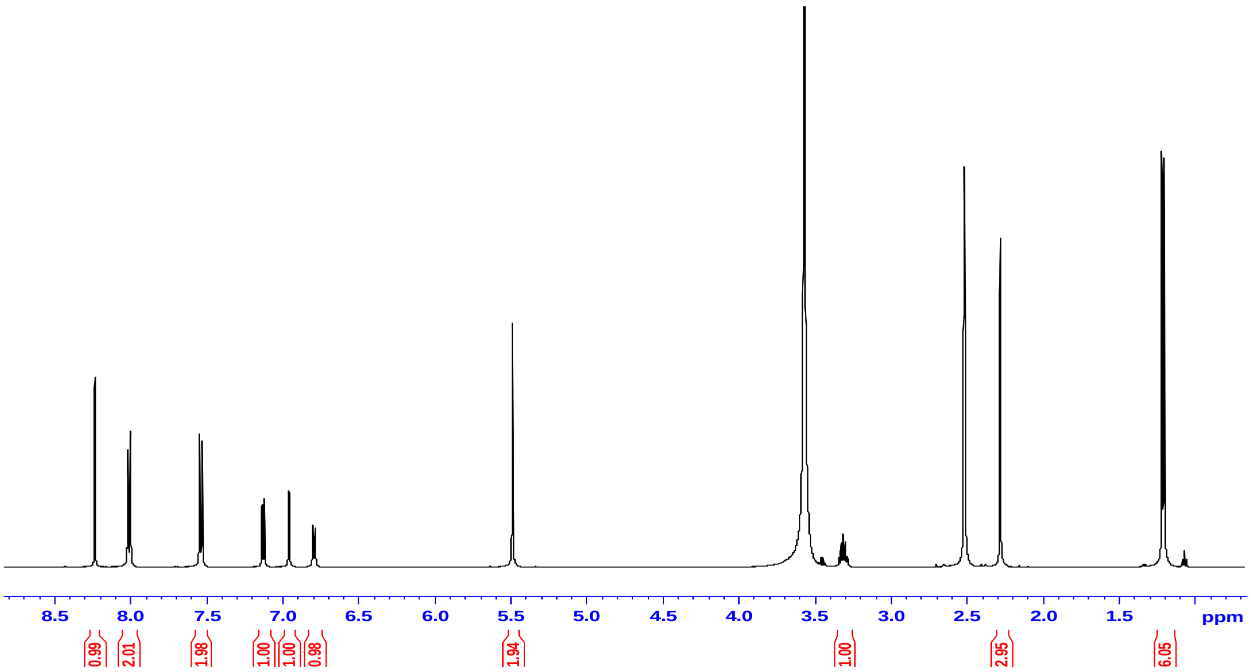

Figure 2. ^{1}H-NMR spectra of compound **4b**

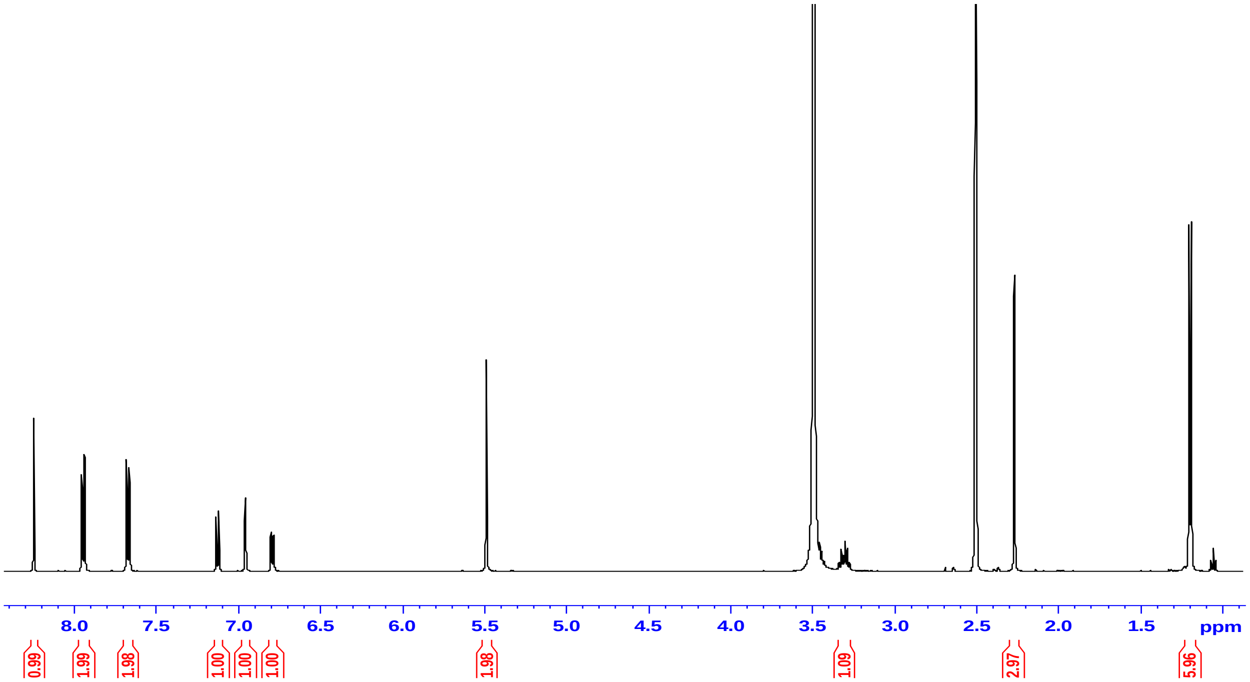

Figure 3. ^{1}H-NMR spectra of compound **4c**

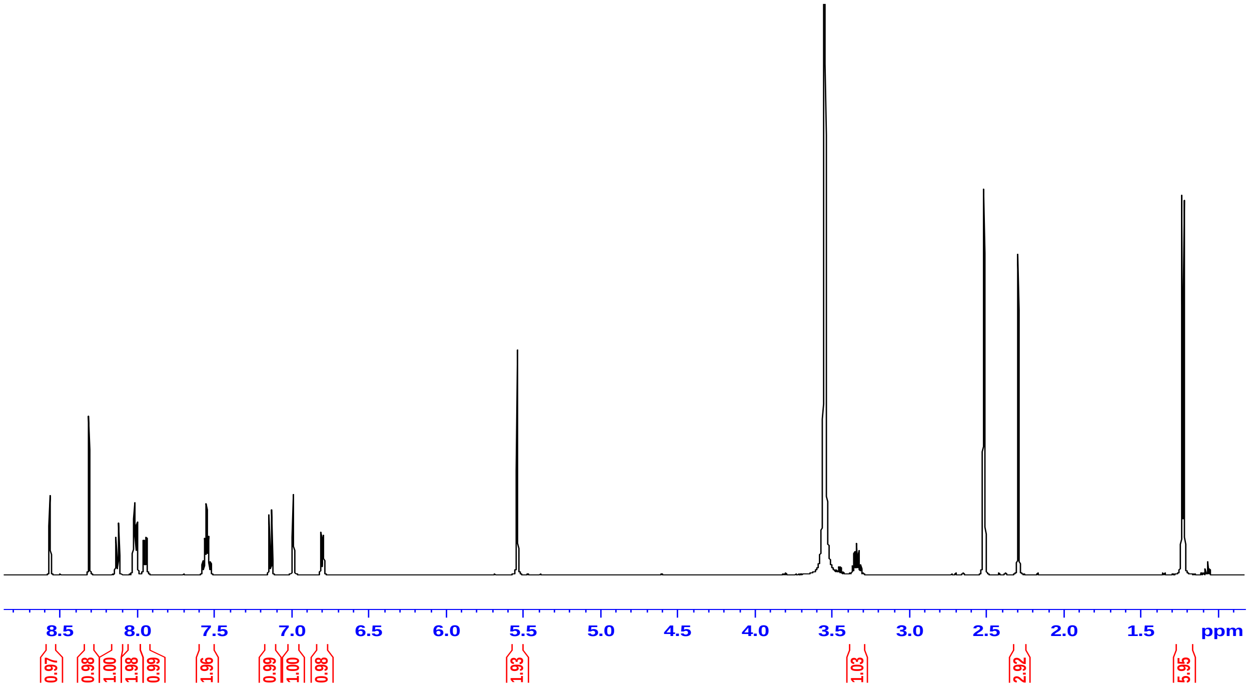

Figure 4. ^{1}H-NMR spectra of compound **4d**

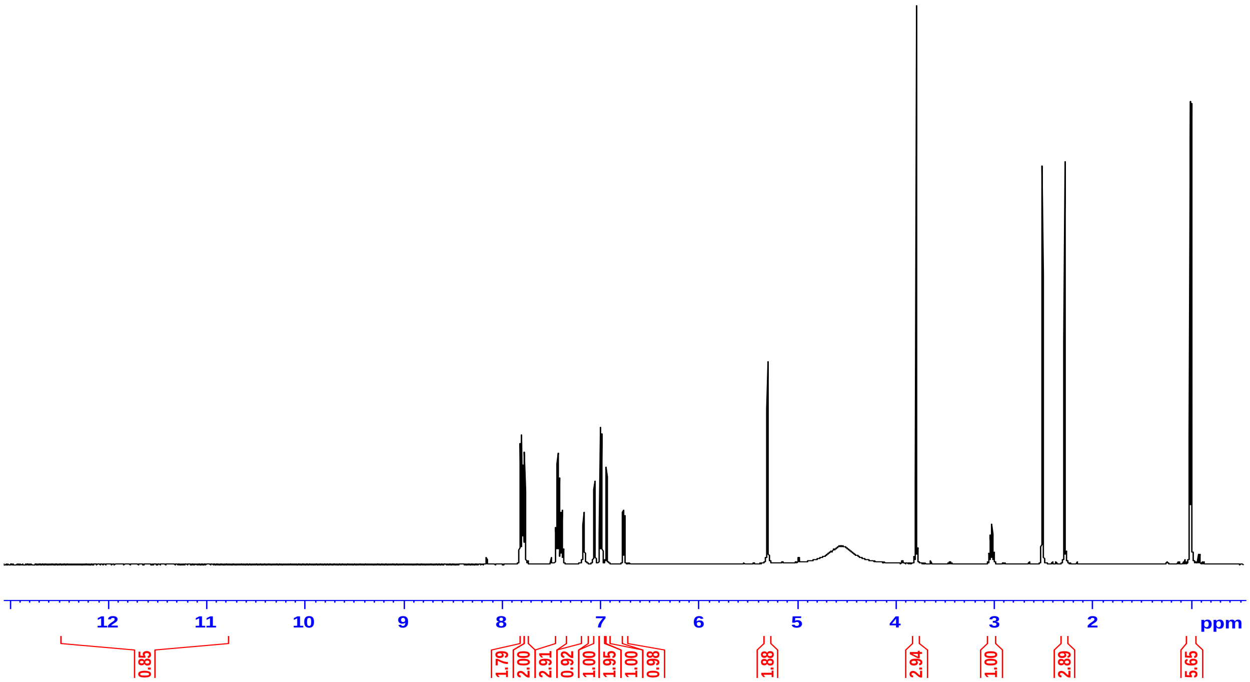

Figure 5. ¹H-NMR spectra of compound **7a**

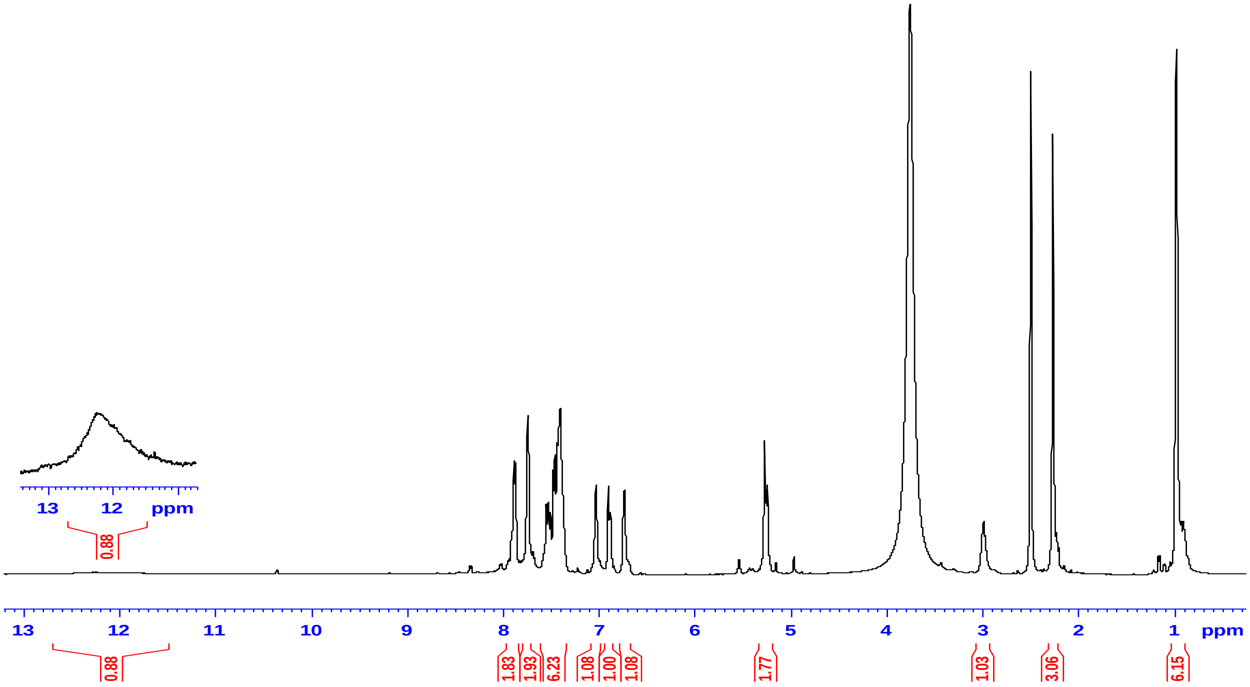

Figure 6. ¹H-NMR spectra of compound **7b**

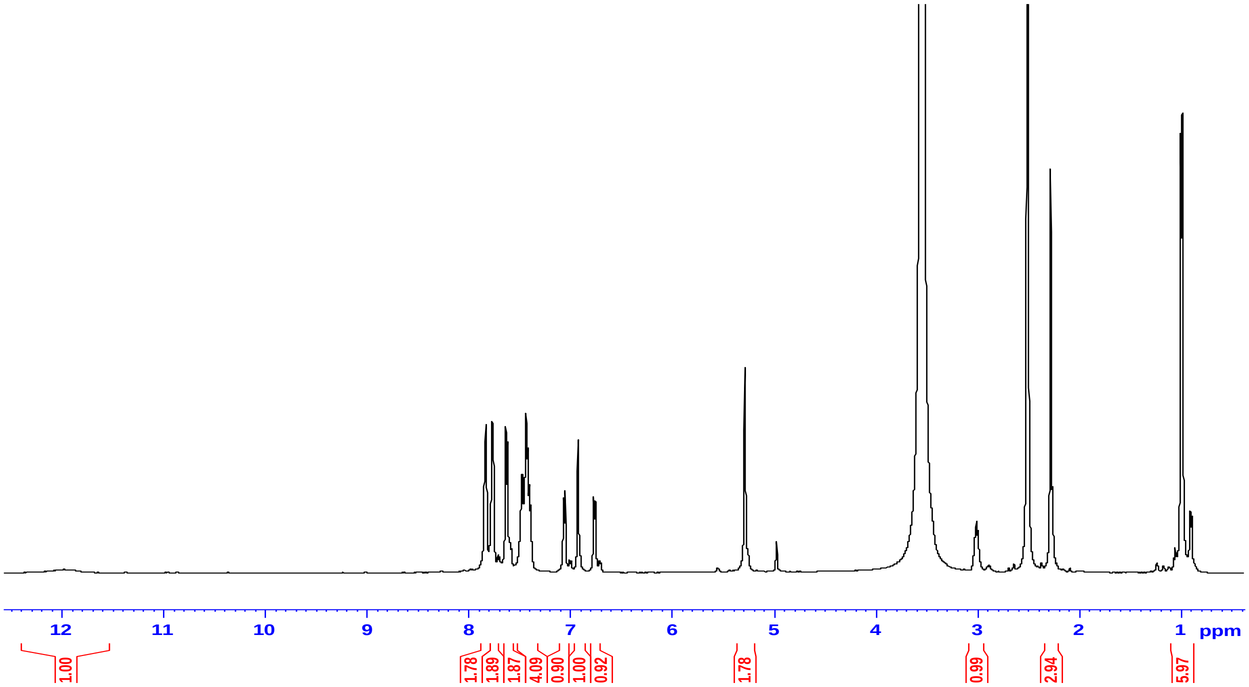

Figure 7. ^{1}H-NMR spectra of compound **7c**

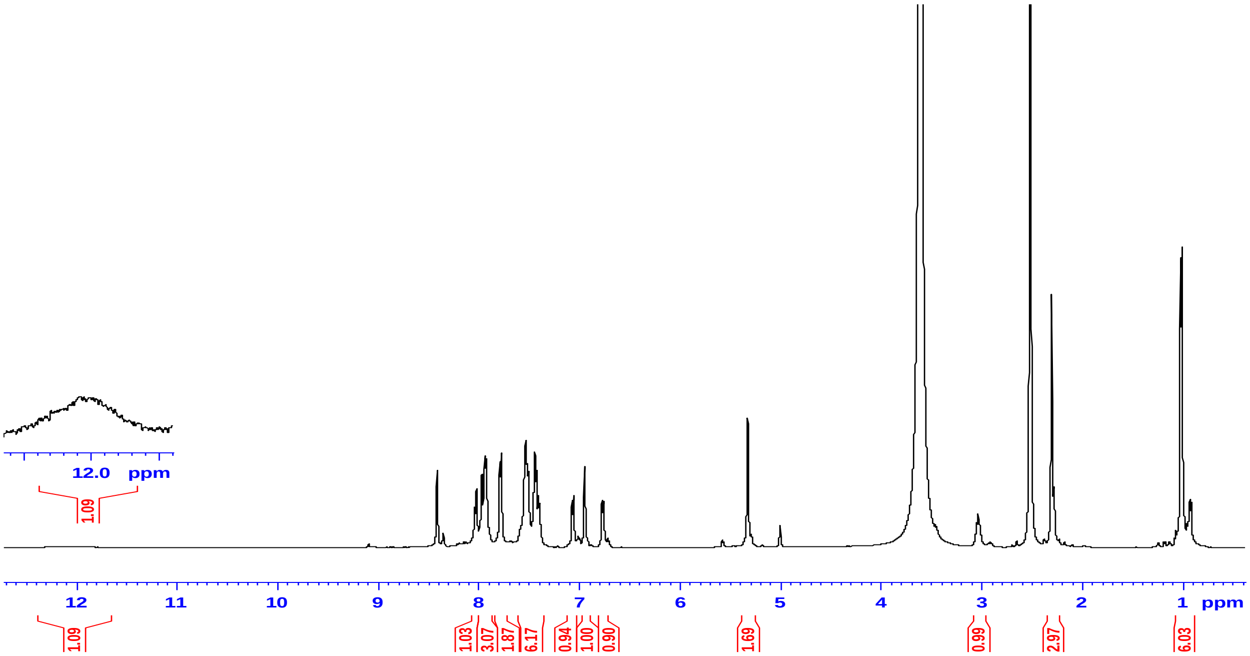

Figure 8. ^{1}H-NMR spectra of compound **7d**

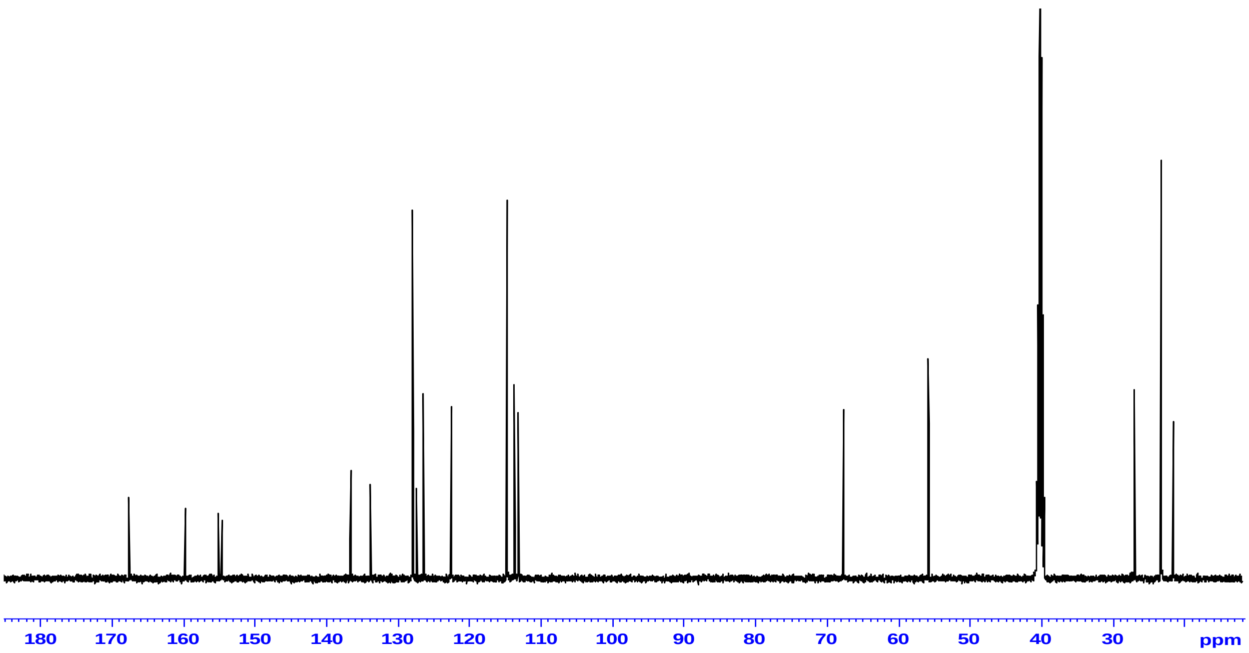

Figure 9. ^{13}C-NMR spectra of compound **4a**

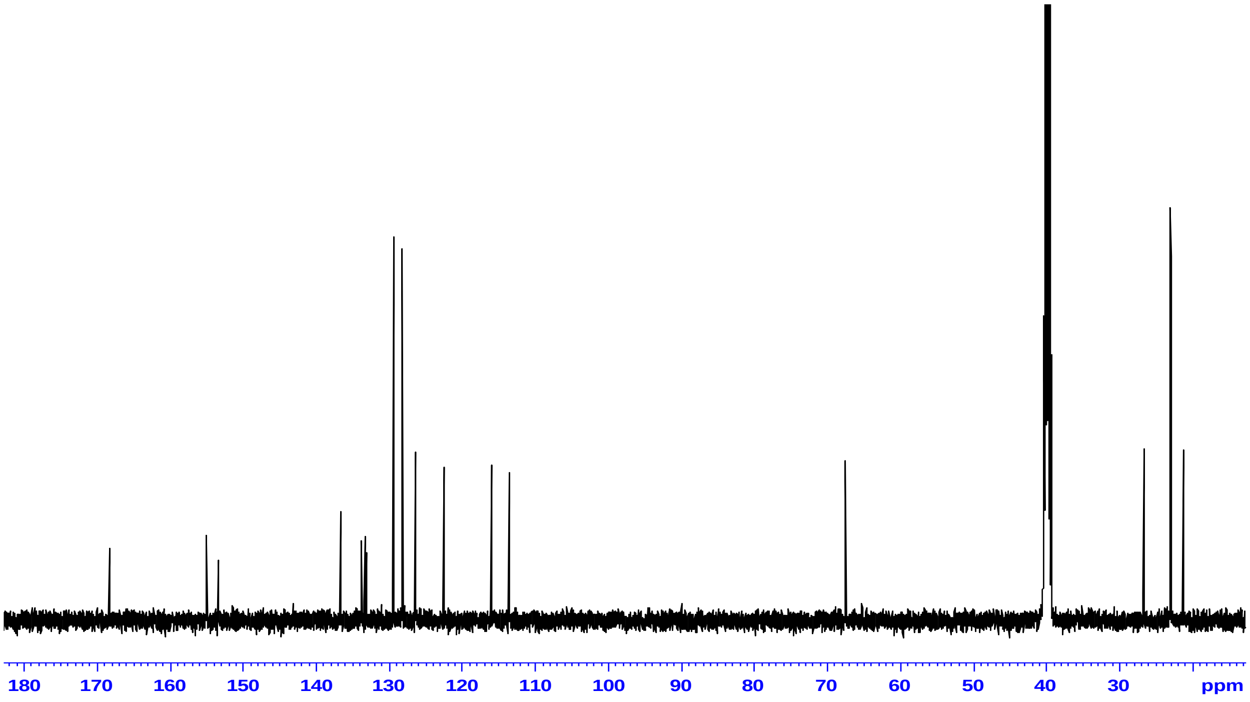

Figure 10. ^{13}C-NMR spectra of compound **4b**

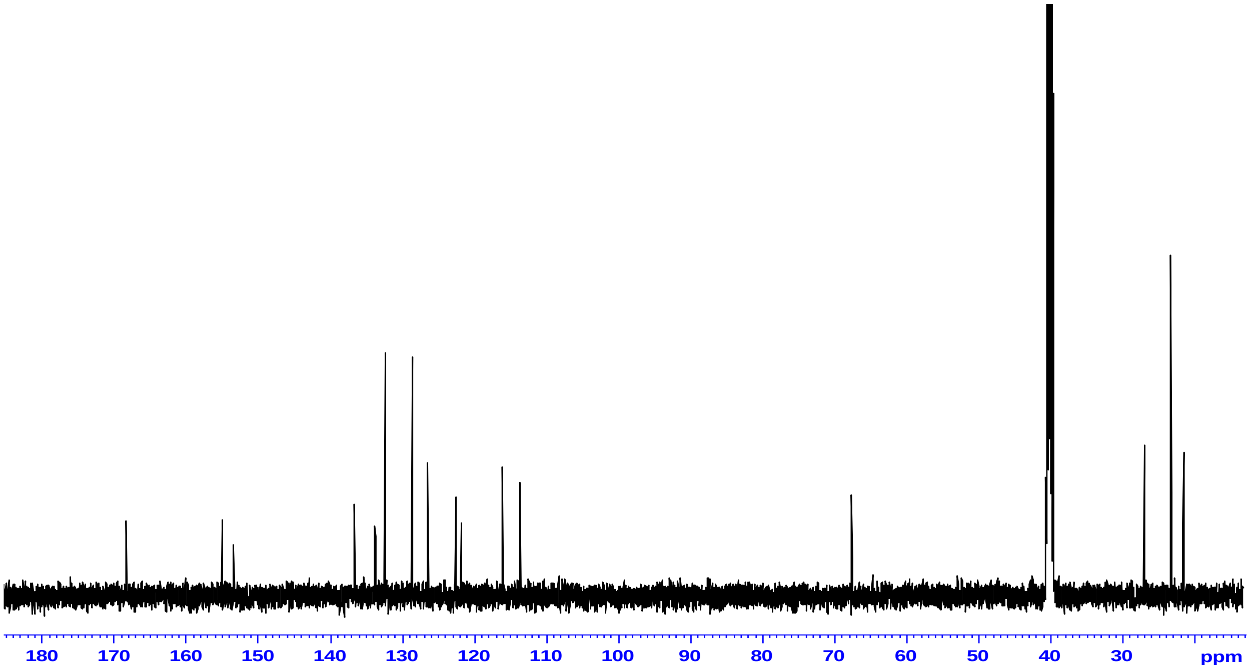

Figure 11. ^{13}C-NMR spectra of compound **4c**

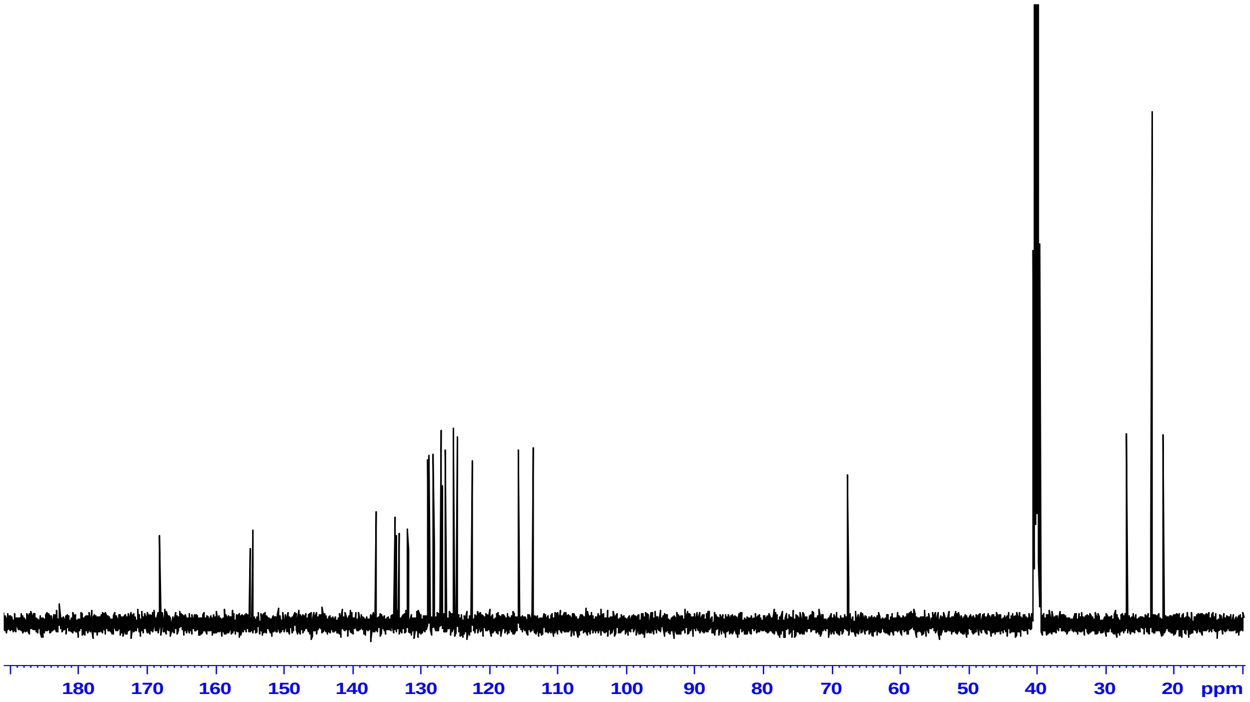

Figure 12. ^{13}C-NMR spectra of compound **4d**

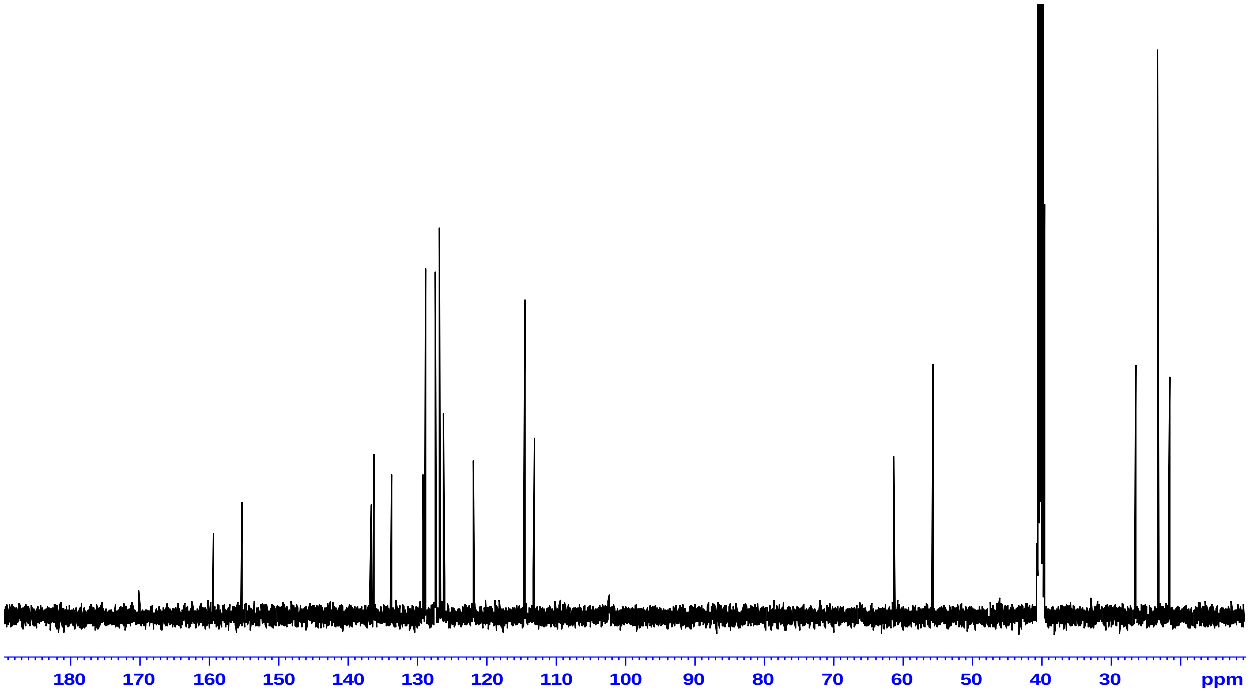

Figure 13. ^{13}C-NMR spectra of compound **7a**

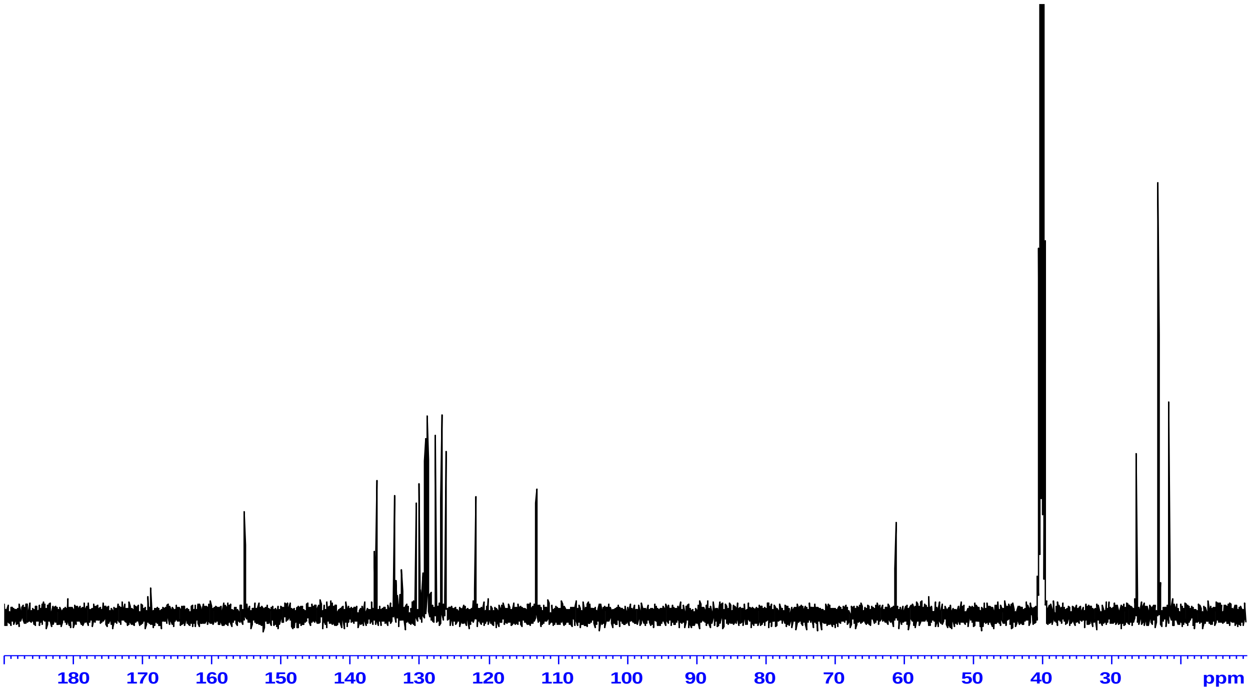

Figure 14. ^{13}C-NMR spectra of compound **7b**

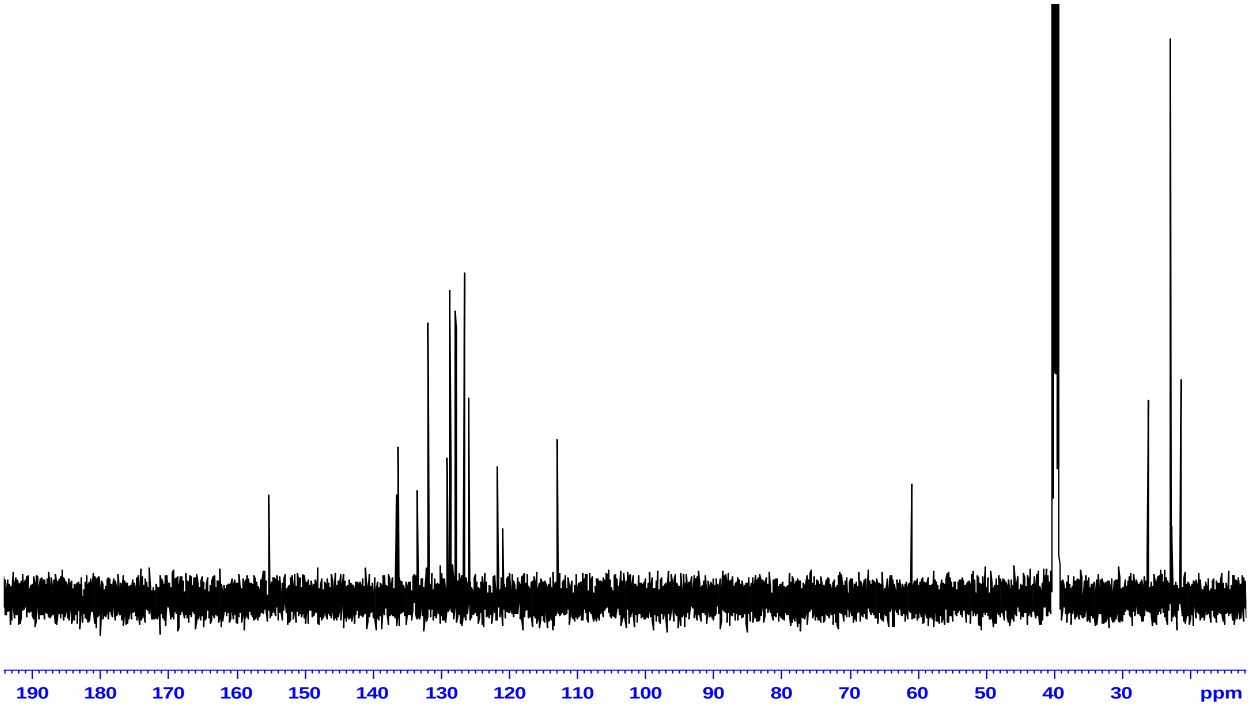

Figure 15. ^{13}C-NMR spectra of compound **7c**

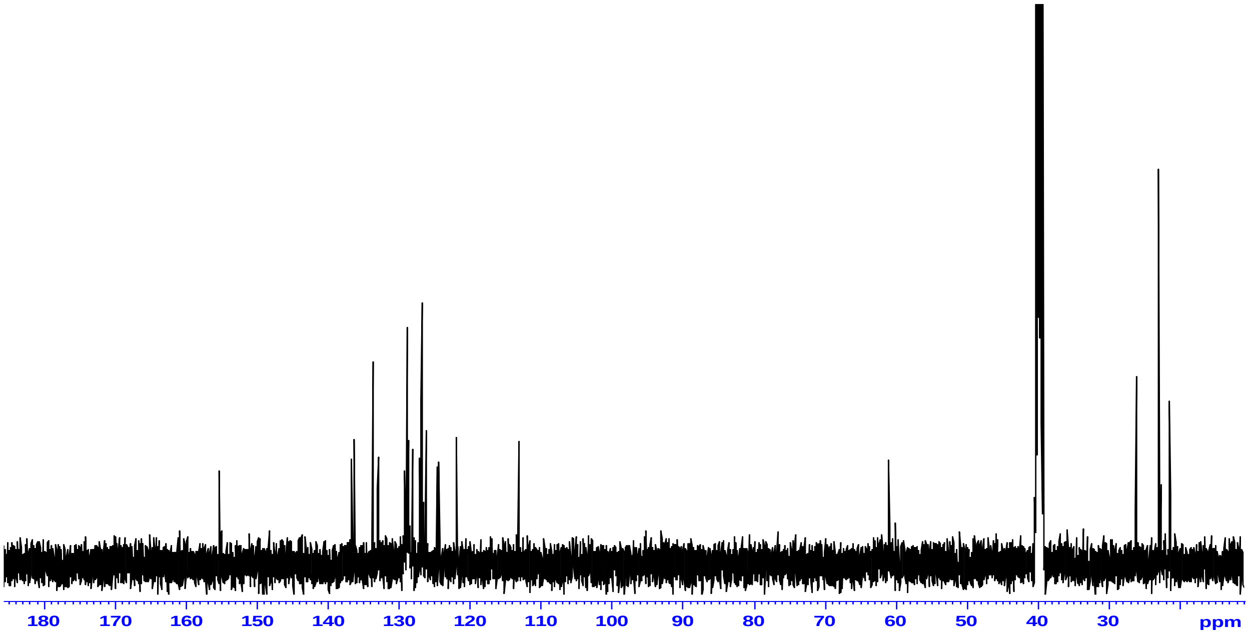

Figure 16. ^{13}C-NMR spectra of compound **7d**

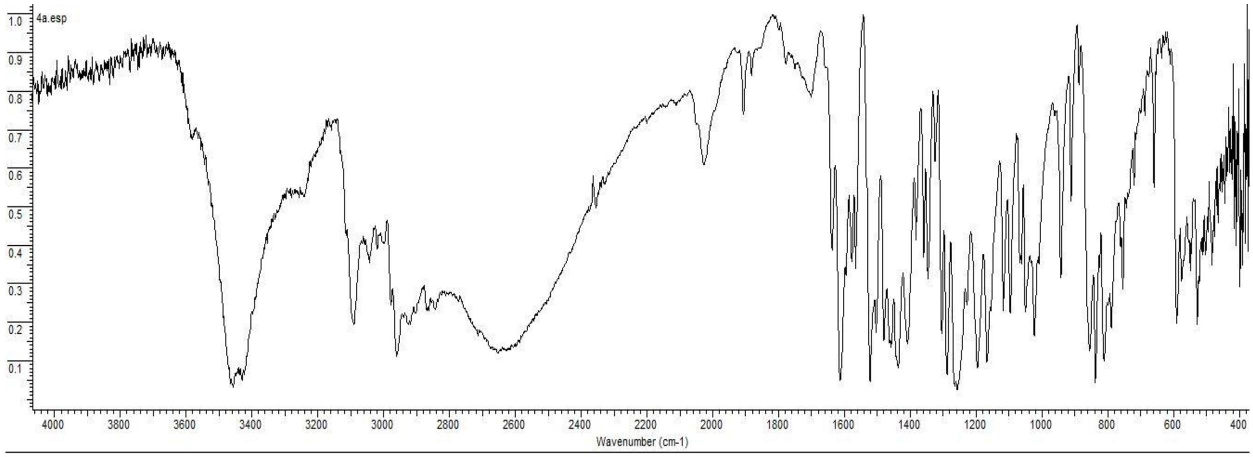

Figure 17. FT-IR spectra of compound **4a**

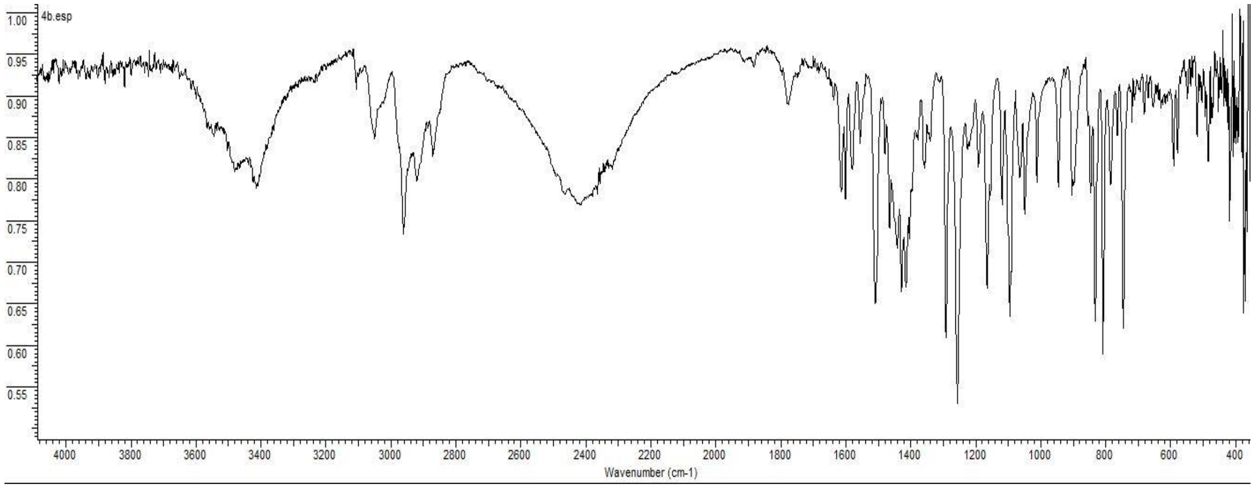

Figure 18. FT-IR spectra of compound **4b**

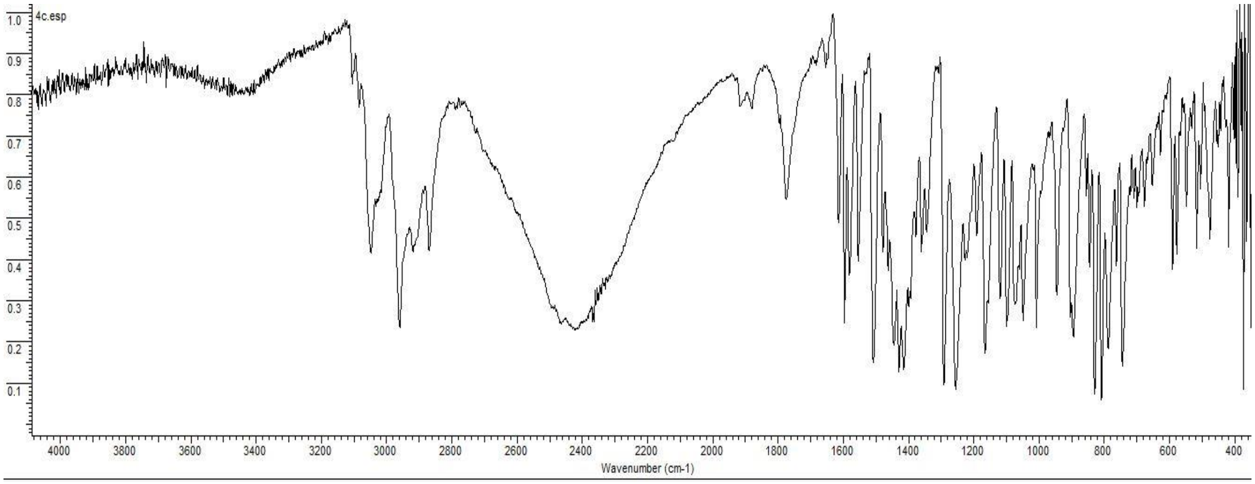

Figure 19. FT-IR spectra of compound **4c**

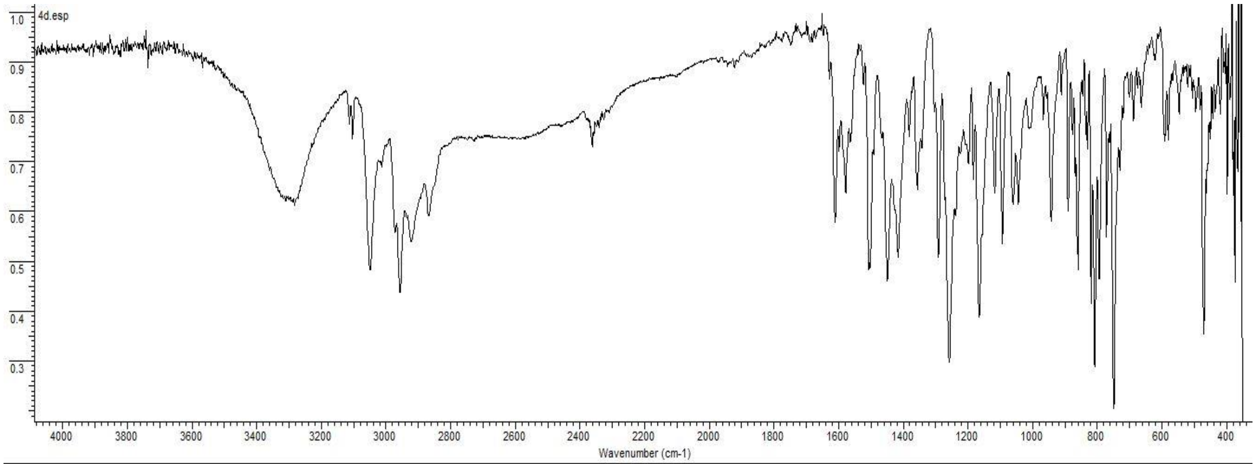

Figure 20. FT-IR spectra of compound **4d**

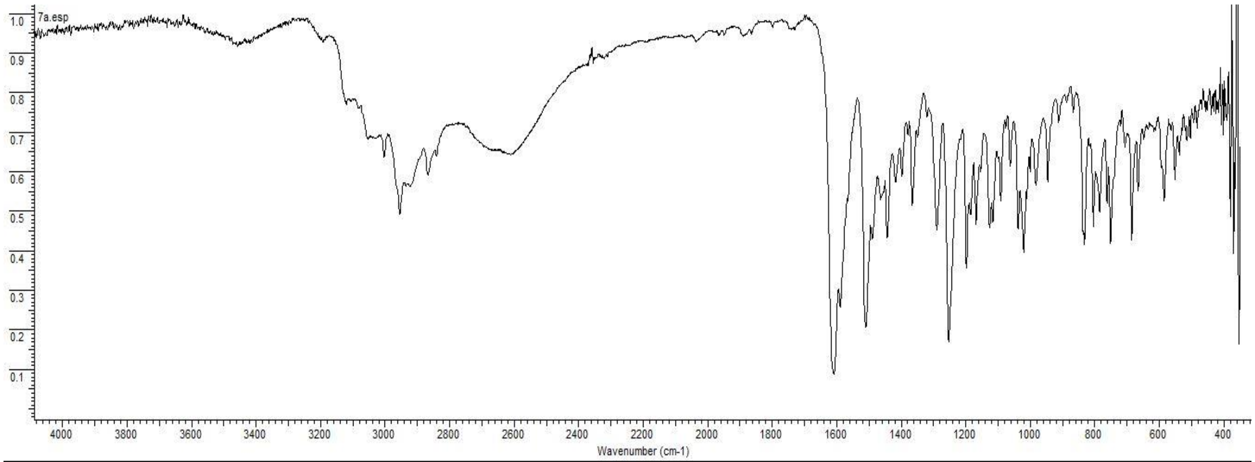

Figure 21. FT-IR spectra of compound **7a**

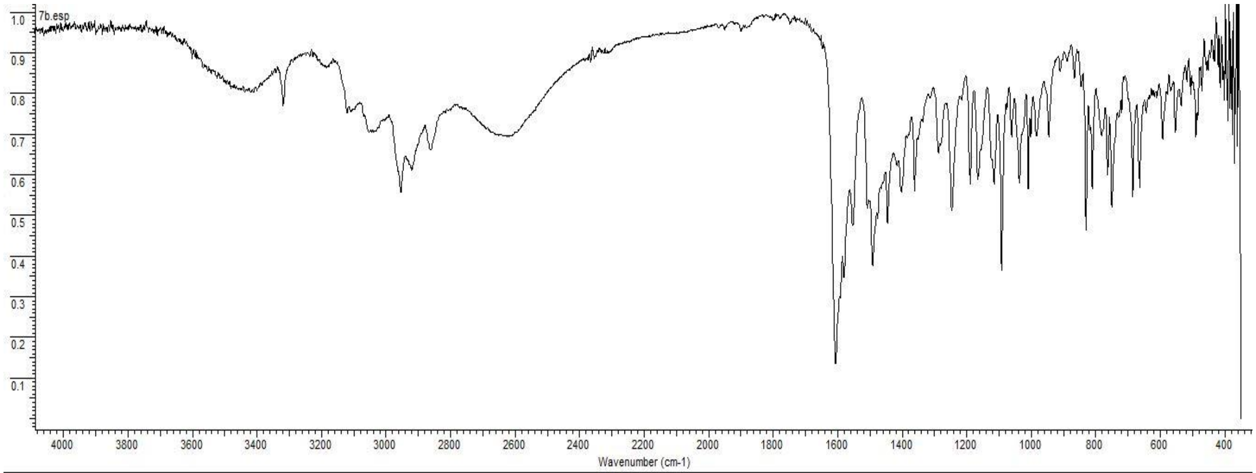

Figure 22. FT-IR spectra of compound **7b**

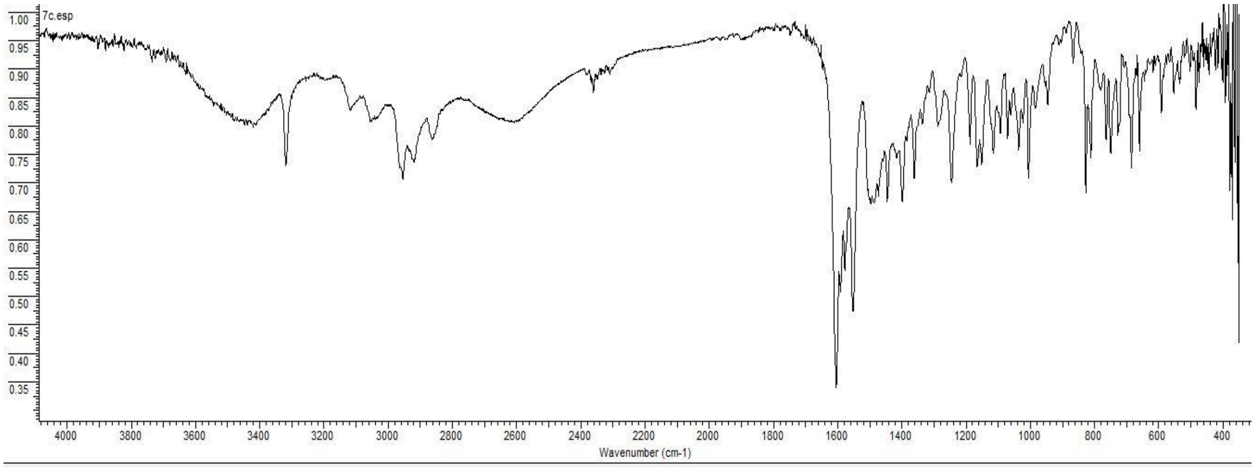

Figure 23. FT-IR spectra of compound **7c**

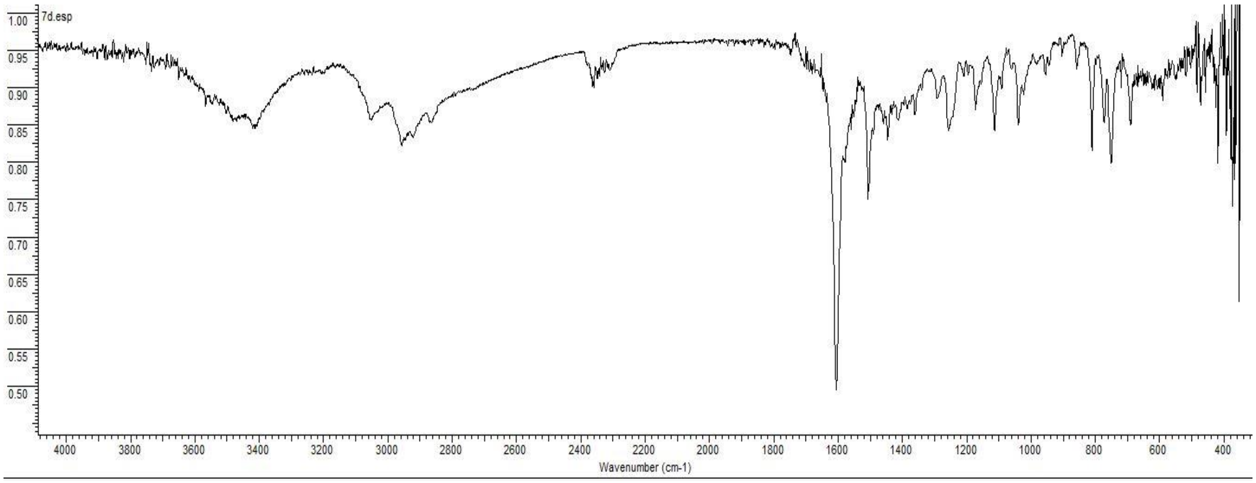

Figure 24. FT-IR spectra of compound **7d**

Acquisition Parameter:

Mass Range Mode	Std/Normal	Trap Drive	43.0	Scan Begin	105 m/z	Auto MS/MS	off
Ion Polarity	Positive	Octopole RF Amplitude	75.0 Vpp	Scan End	500 m/z		
Ion Source Type	ESI			Averages	1 Spectra		
Dry Temp (Set)	350 °C	Capillary Exit	95.5 Volt	Max. Accu Time	100000 µs		
Nebulizer (Set)	60.00 psi	Skimmer	40.0 Volt	ICC Target	40000		
Dry Gas (Set)	12.00 l/min	Oct 1 DC	12.00 Volt	Charge Control	on		
		Oct 2 DC	1.70 Volt				

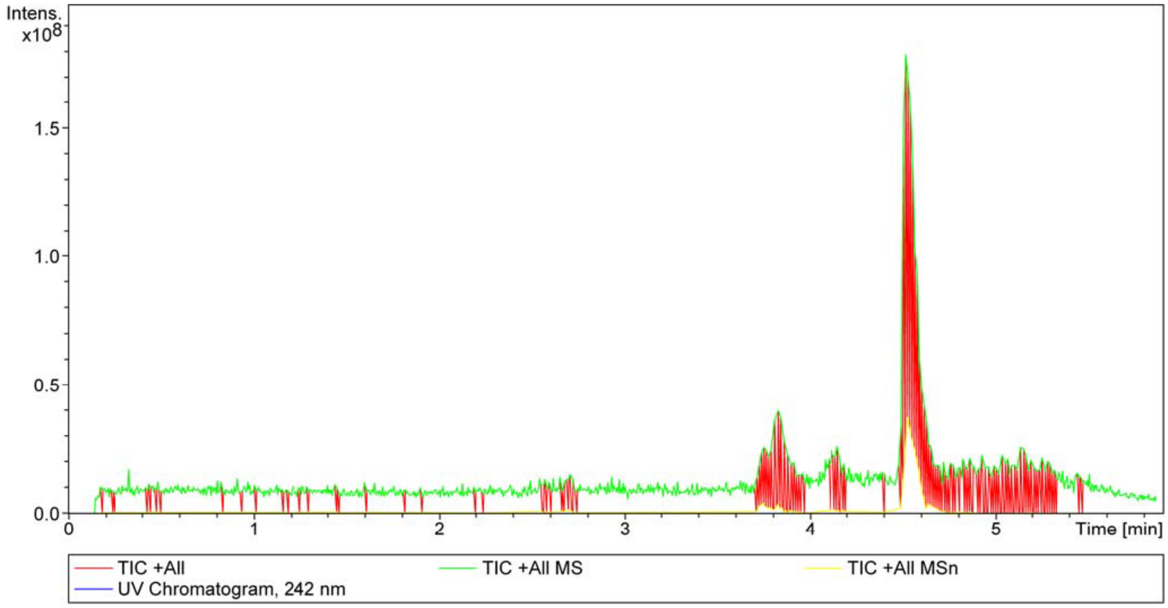

Compound List:

#	RT [min]	Range [min]	MS(n) Isol. m/z
n.a.	4.5	4.5	
n.a.	4.5	4.5	354.5

+MS, 4.5min #882

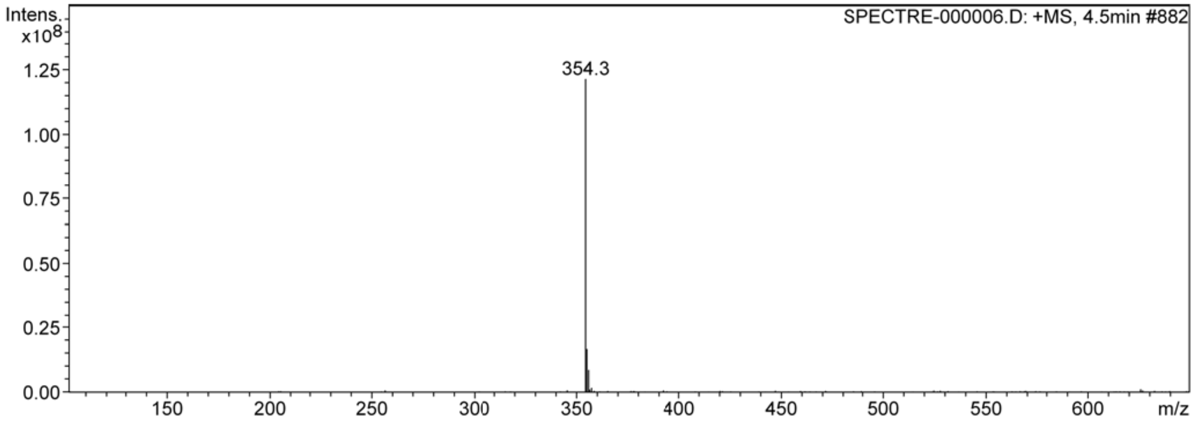

Component	Molecular Mass	Molecule	Absolute Abundance	Relative Abundance

Figure 25. ESI-MS spectrum of compound **4a**

Acquisition Parameter:

Mass Range Mode	Std/Normal	Trap Drive	43.0	Scan Begin	105 m/z	Auto MS/MS	off
Ion Polarity	Positive	Octopole RF Amplitude	75.0 Vpp	Scan End	500 m/z		
Ion Source Type	ESI			Averages	1 Spectra		
Dry Temp (Set)	350 °C	Capillary Exit	95.5 Volt	Max. Accu Time	100000 µs		
Nebulizer (Set)	60.00 psi	Skimmer	40.0 Volt	ICC Target	40000		
Dry Gas (Set)	12.00 l/min	Oct 1 DC	12.00 Volt	Charge Control	on		
		Oct 2 DC	1.70 Volt				

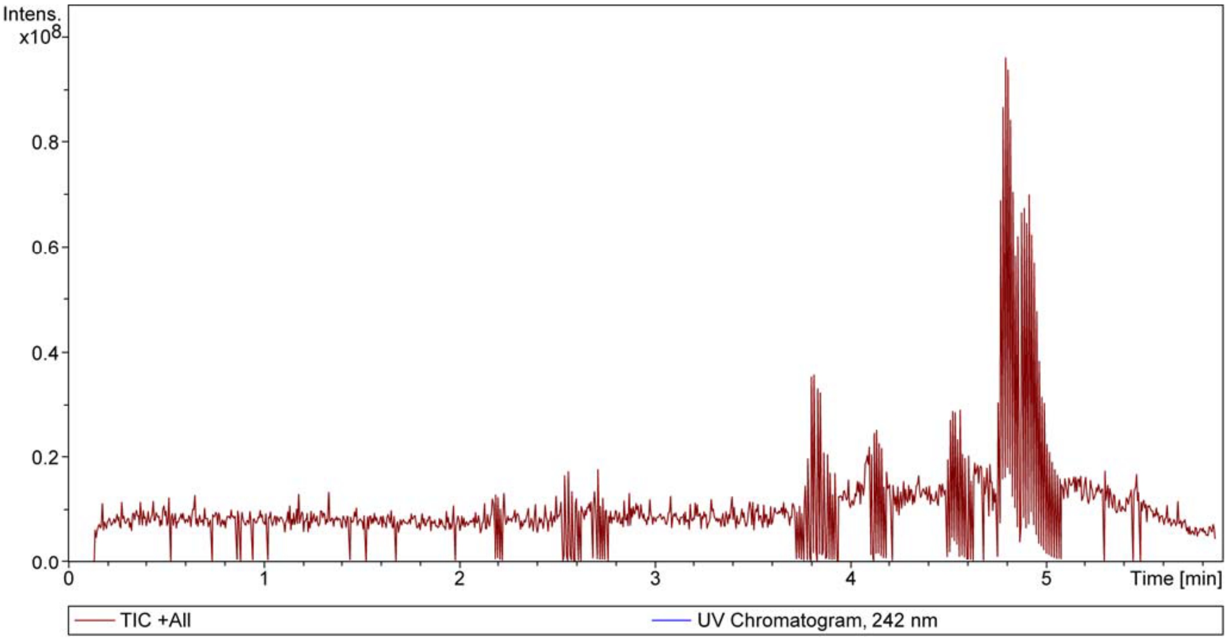

Compound List:

#	RT [min]	Range [min]	MS(n) Isol. m/z
n.a.	4.9	4.9	
n.a.	4.9	4.9	358.9

+MS, 4.9min #948

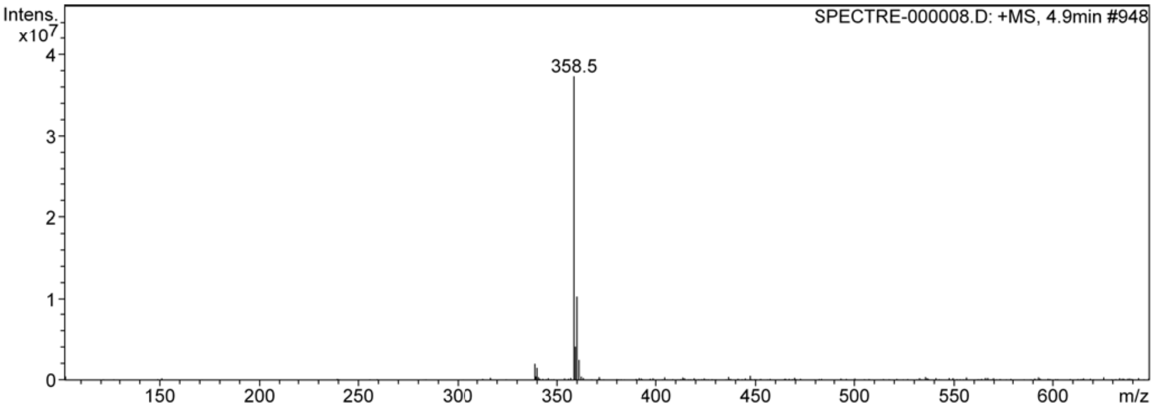

Component	Molecular Mass	Molecule	Absolute Abundance	Relative Abundance

Figure 26. ESI-MS spectrum of compound **4b**

Acquisition Parameter:

Mass Range Mode	Std/Normal	Trap Drive	43.0	Scan Begin	105 m/z	Auto MS/MS off
Ion Polarity	Positive	Octopole RF Amplitude	75.0 Vpp	Scan End	500 m/z	
Ion Source Type	ESI			Averages	1 Spectra	
Dry Temp (Set)	350 °C	Capillary Exit	95.5 Volt	Max. Accu Time	100000 µs	
Nebulizer (Set)	60.00 psi	Skimmer	40.0 Volt	ICC Target	40000	
Dry Gas (Set)	12.00 l/min	Oct 1 DC	12.00 Volt	Charge Control	on	
		Oct 2 DC	1.70 Volt			

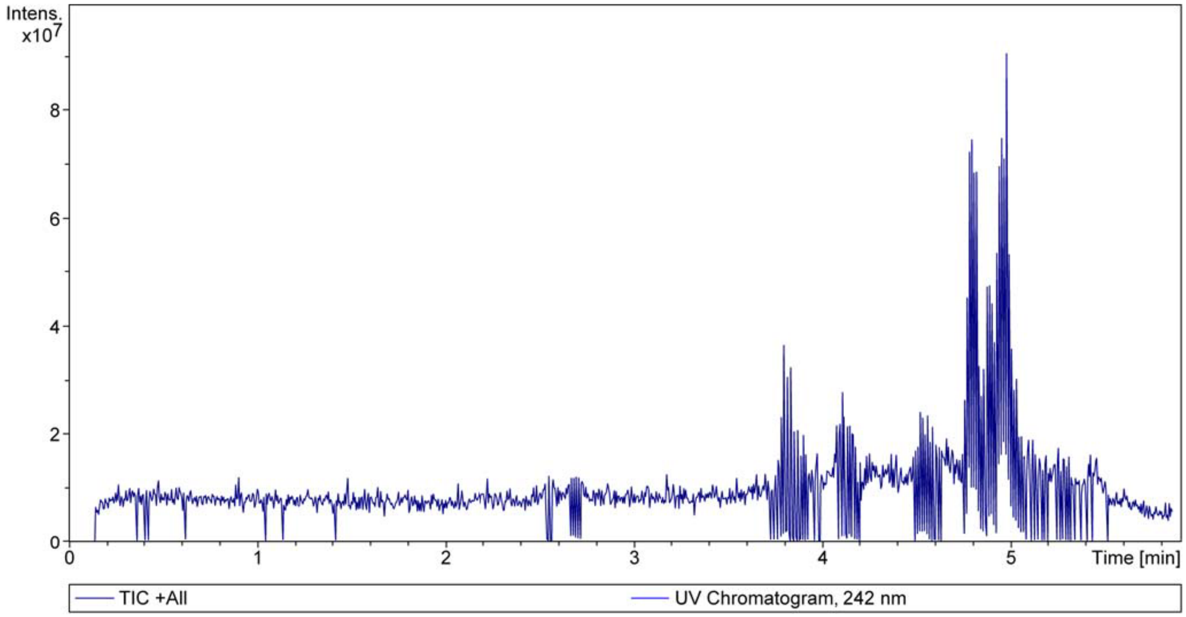

Compound List:

#	RT [min]	Range [min]	MS(n) Isol. m/z
n.a.	5.0	5.0	
n.a.	5.0	5.0	403.4

+MS, 5.0min #965

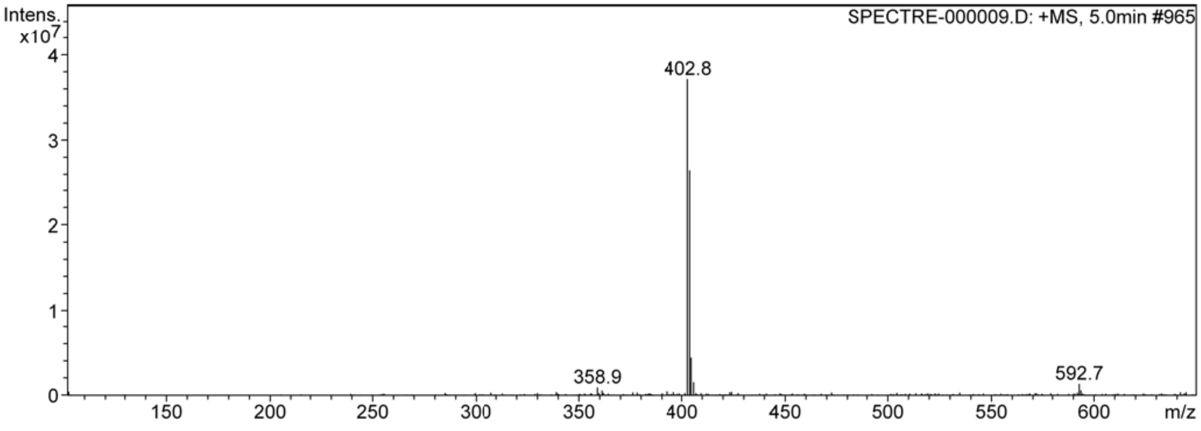

Component	Molecular Mass	Molecule	Absolute Abundance	Relative Abundance

Figure 27. ESI-MS spectrum of compound **4c**

Acquisition Parameter:

Mass Range Mode	Std/Normal	Trap Drive	43.0	Scan Begin	105 m/z	Auto MS/MS	off
Ion Polarity	Positive	Octopole RF Amplitude	75.0 Vpp	Scan End	500 m/z		
Ion Source Type	ESI			Averages	1 Spectra		
Dry Temp (Set)	350 °C	Capillary Exit	95.5 Volt	Max. Accu Time	100000 µs		
Nebulizer (Set)	60.00 psi	Skimmer	40.0 Volt	ICC Target	40000		
Dry Gas (Set)	12.00 l/min	Oct 1 DC	12.00 Volt	Charge Control	on		
		Oct 2 DC	1.70 Volt				

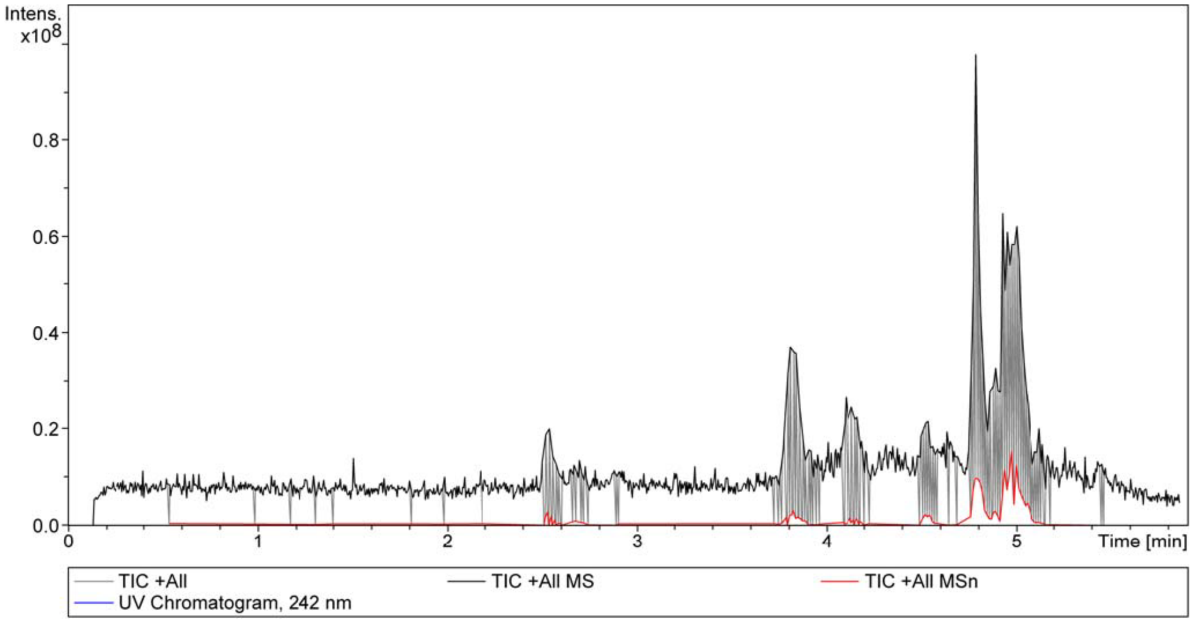

Compound List:

#	RT [min]	Range [min]	MS(n) Isol. m/z
n.a.	5.0	5.0	
n.a.	5.0	5.0	374.5

+MS, 5.0min #967

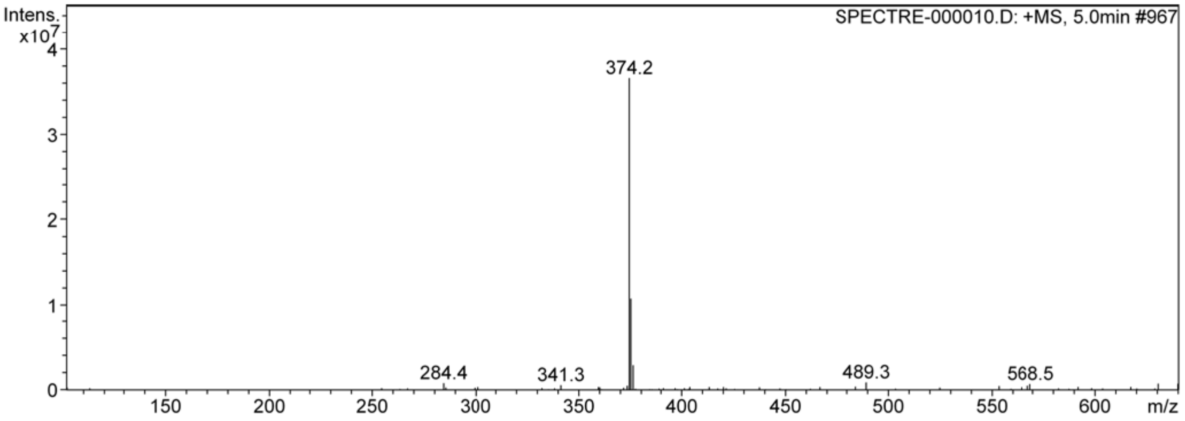

Component	Molecular Mass	Molecule	Absolute Abundance	Relative Abundance

Figure 28. ESI-MS spectrum of compound **4d**

Acquisition Parameter:

Mass Range Mode	Std/Normal	Trap Drive	43.0	Scan Begin	105 m/z	Auto MS/MS off
Ion Polarity	Positive	Octopole RF Amplitude	75.0 Vpp	Scan End	500 m/z	
Ion Source Type	ESI			Averages	1 Spectra	
Dry Temp (Set)	350 °C	Capillary Exit	95.5 Volt	Max. Accu Time	100000 µs	
Nebulizer (Set)	60.00 psi	Skimmer	40.0 Volt	ICC Target	40000	
Dry Gas (Set)	12.00 l/min	Oct 1 DC	12.00 Volt	Charge Control	on	
		Oct 2 DC	1.70 Volt			

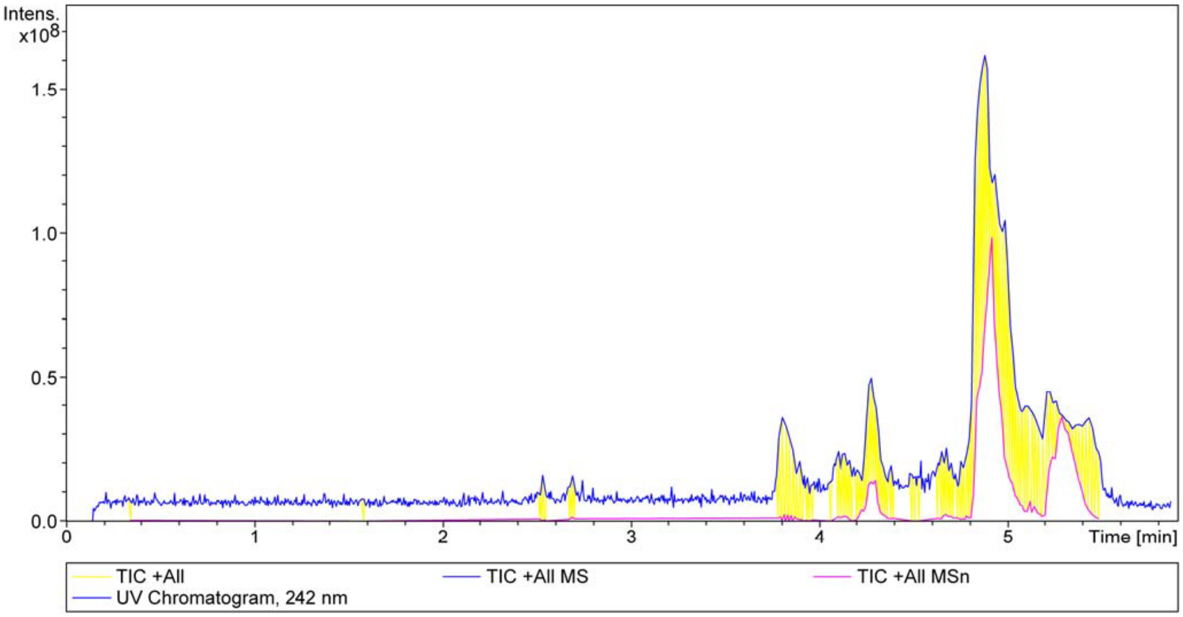

Compound List:

#	RT [min]	Range [min]	MS(n) Isol. m/z
n.a.	4.9	4.9	
n.a.	4.9	4.9	472.7

+MS, 4.9min #943

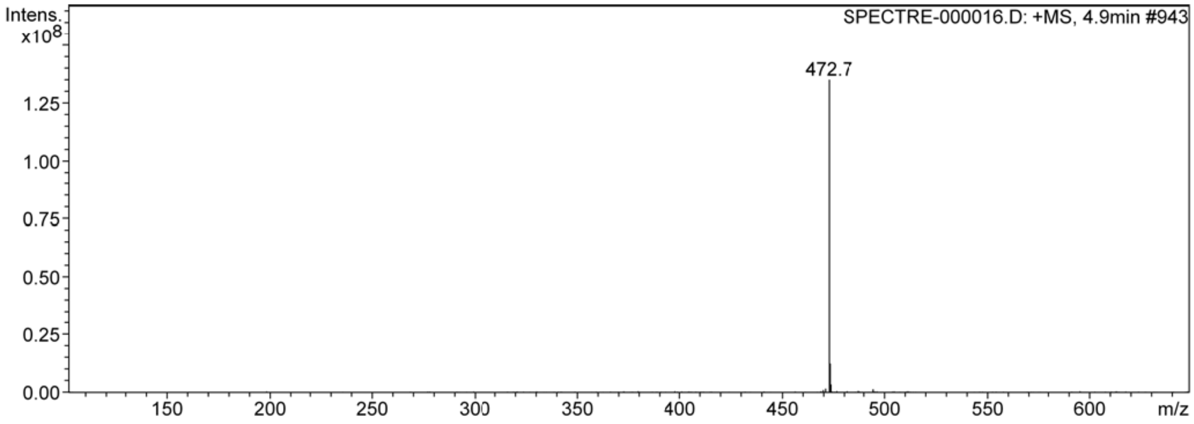

Component	Molecular Mass	Molecule	Absolute Abundance	Relative Abundance

Figure 29. ESI-MS spectrum of compound **7a**

Acquisition Parameter:

Mass Range Mode	Std/Normal	Trap Drive	43.0	Scan Begin	105 m/z	Auto MS/MS off
Ion Polarity	Positive	Octopole RF Amplitude	75.0 Vpp	Scan End	500 m/z	
Ion Source Type	ESI			Averages	1 Spectra	
Dry Temp (Set)	350 °C	Capillary Exit	95.5 Volt	Max. Accu Time	100000 µs	
Nebulizer (Set)	60.00 psi	Skimmer	40.0 Volt	ICC Target	40000	
Dry Gas (Set)	12.00 l/min	Oct 1 DC	12.00 Volt	Charge Control	on	
		Oct 2 DC	1.70 Volt			

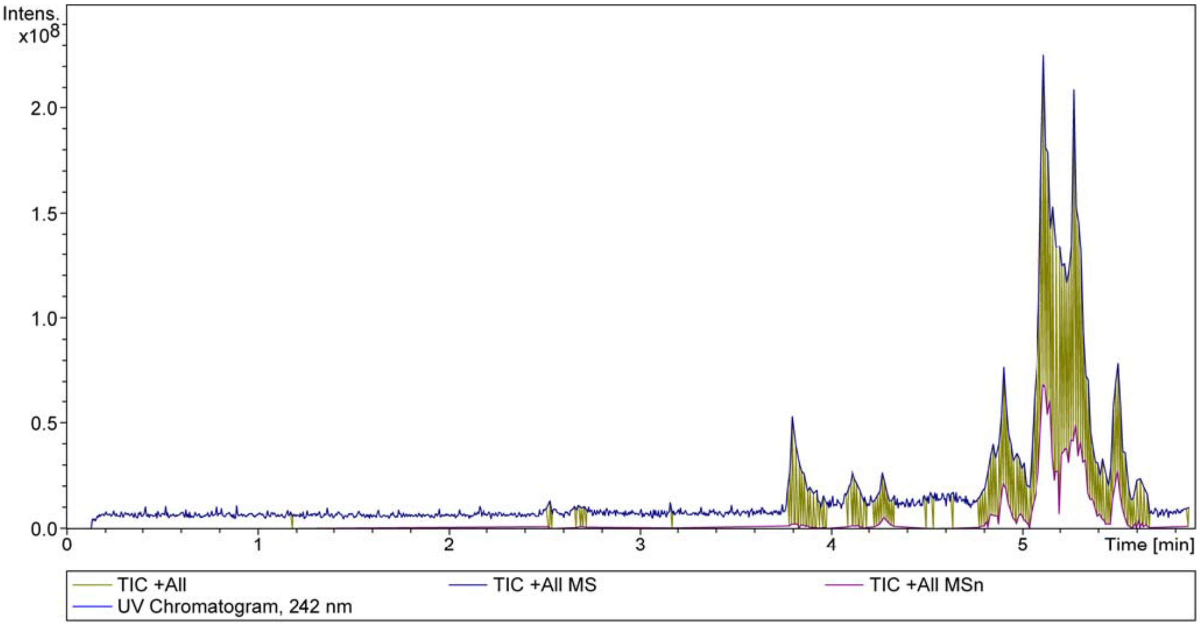

Compound List:

#	RT [min]	Range [min]	MS(n) Isol. m/z
n.a.	5.3	5.3	
n.a.	5.3	5.3	476.8

+MS, 5.3min #1014

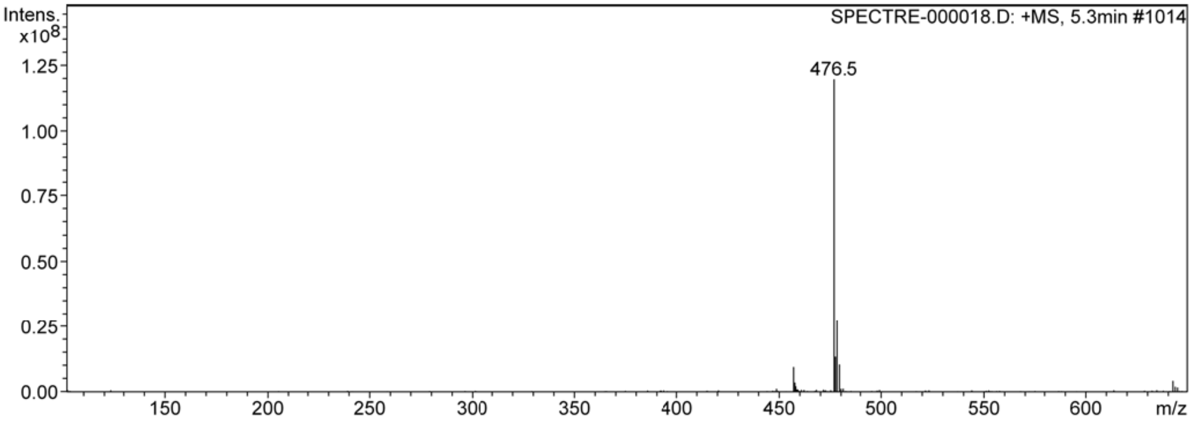

Component	Molecular Mass	Molecule	Absolute Abundance	Relative Abundance

Figure 30. ESI-MS spectrum of compound **7b**

Acquisition Parameter:

Mass Range Mode	Std/Normal	Trap Drive	43.0	Scan Begin	105 m/z	Auto MS/MS	off
Ion Polarity	Positive	Octopole RF Amplitude	75.0 Vpp	Scan End	500 m/z		
Ion Source Type	ESI			Averages	1 Spectra		
Dry Temp (Set)	350 °C	Capillary Exit	95.5 Volt	Max. Accu Time	100000 µs		
Nebulizer (Set)	60.00 psi	Skimmer	40.0 Volt	ICC Target	40000		
Dry Gas (Set)	12.00 l/min	Oct 1 DC	12.00 Volt	Charge Control	on		
		Oct 2 DC	1.70 Volt				

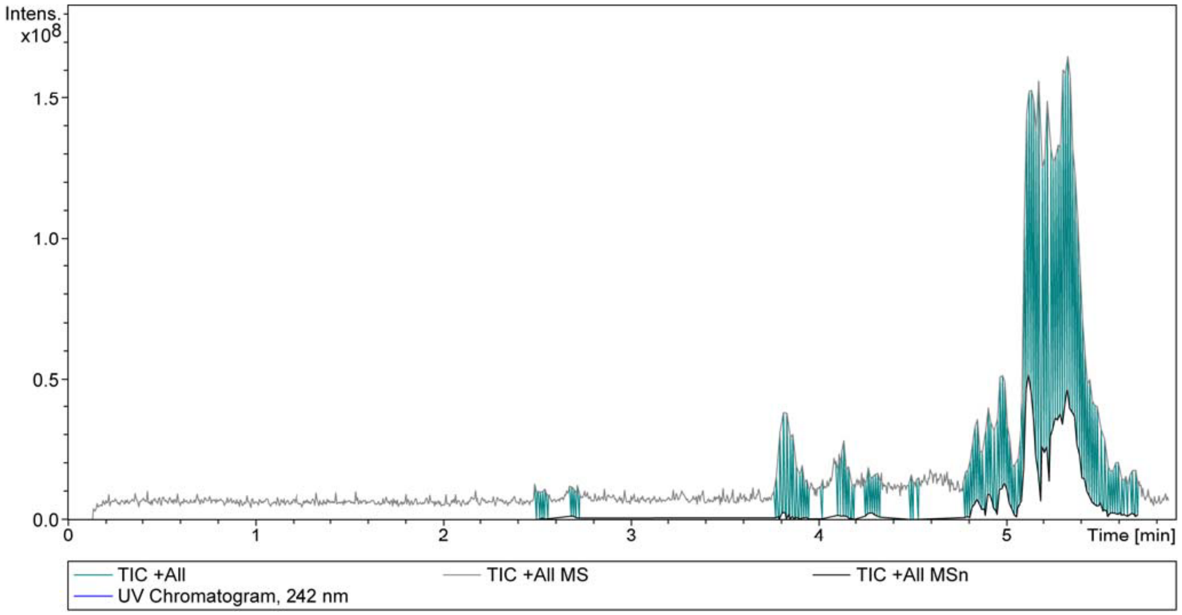

Compound List:

#	RT [min]	Range [min]	MS(n) Isol. m/z
n.a.	5.3	5.3	
n.a.	5.3	5.3	521.4

+MS, 5.3min #1021

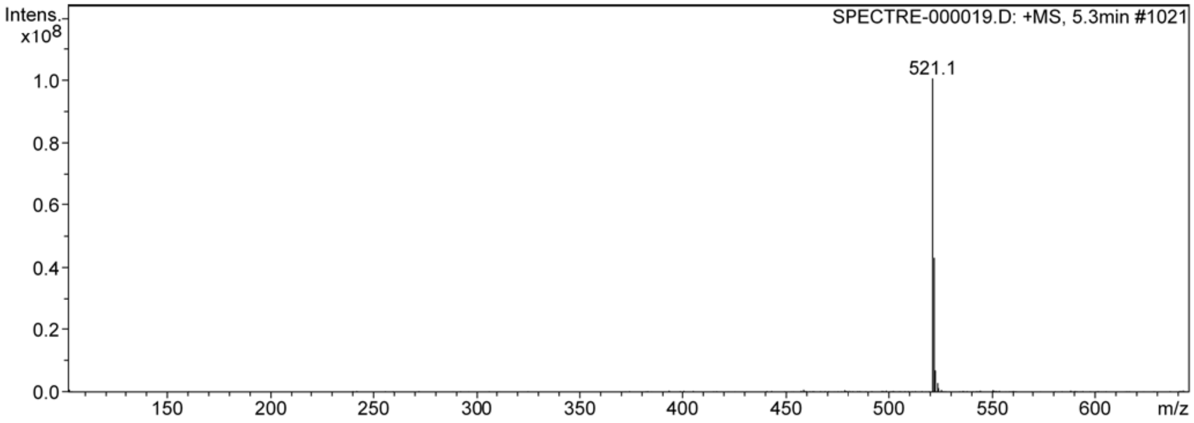

Component	Molecular Mass	Molecule	Absolute Abundance	Relative Abundance

Figure 31. ESI-MS spectrum of compound **7c**

Acquisition Parameter:

Mass Range Mode	Std/Normal	Trap Drive	43.0	Scan Begin	105 m/z	Auto MS/MS off
Ion Polarity	Positive	Octopole RF Amplitude	75.0 Vpp	Scan End	500 m/z	
Ion Source Type	ESI			Averages	1 Spectra	
Dry Temp (Set)	350 °C	Capillary Exit	95.5 Volt	Max. Accu Time	100000 μs	
Nebulizer (Set)	60.00 psi	Skimmer	40.0 Volt	ICC Target	40000	
Dry Gas (Set)	12.00 l/min	Oct 1 DC	12.00 Volt	Charge Control	on	
		Oct 2 DC	1.70 Volt			

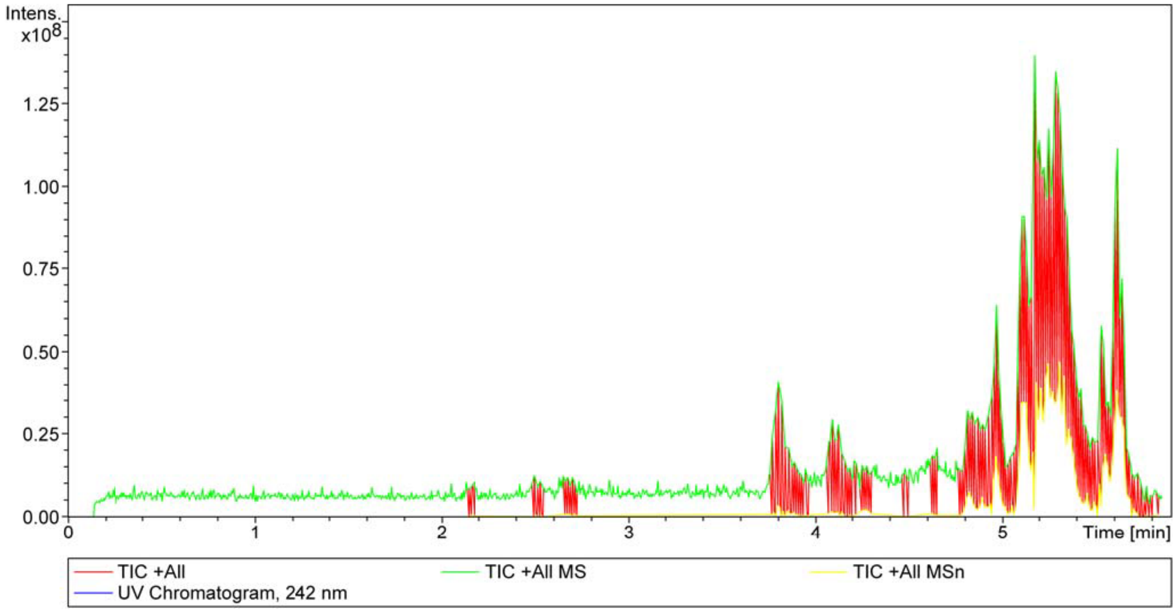

Compound List:

#	RT [min]	Range [min]	MS(n) Isol. m/z
n.a.	5.3	5.3	
n.a.	5.3	5.3	492.6

+MS, 5.3min #1019

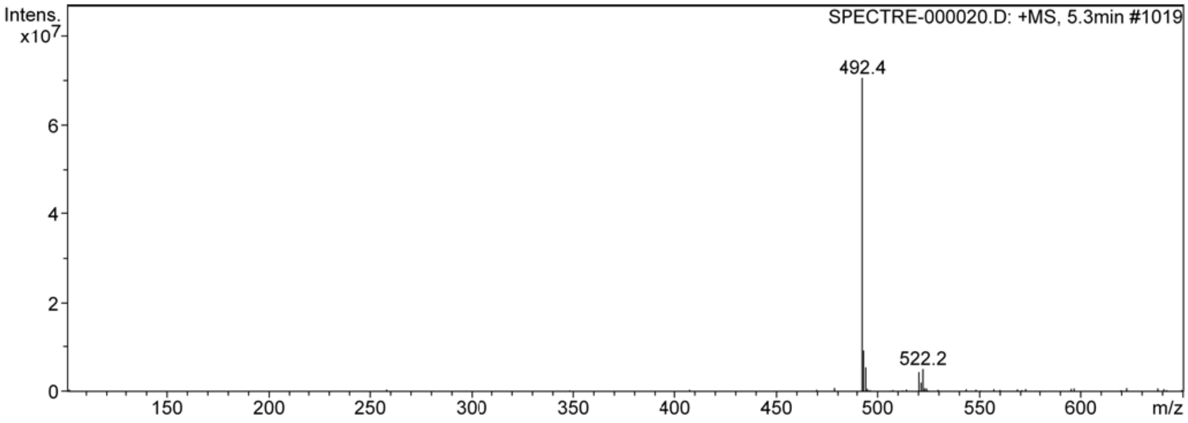

Component	Molecular Mass	Molecule	Absolute Abundance	Relative Abundance

Figure 32. ESI-MS spectrum of compound **7d**
